# Telmisartan ameliorates reproductive dysfunction, oxidative stress, and mitochondrial damages induced by fipronil in male rats via regulation of Nrf2/HO-1/PGC-1*α*/MNF2/DRP1

**DOI:** 10.1007/s00210-025-04812-6

**Published:** 2025-12-19

**Authors:** Alyaa R. Salama, Asmaa A. Aboushouk, Ali El-Far, Neveen R. Ashoura, Aya H. Rohiem, Hanan A. Edres, Hebatallah M. Saad

**Affiliations:** 1 Department of Pathology, Faculty of Veterinary Medicine, Alexandria University, Alexandria, Egypt; 2 Department of Biochemistry, Faculty of Veterinary Medicine, Damanhour University, Damanhour, 22511 Egypt; 3 Department of Pharmacology, Faculty of Veterinary Medicine, Alexandria University, Alexandria, Egypt; 4 Department of Physiology, Faculty of Veterinary Medicine, Alexandria University, Alexandria, Egypt; 5 Department of Biochemistry, Faculty of Veterinary Medicine, Alexandria University, Alexandria, Egypt; 6 Department of Pathology, Faculty of Veterinary Medicine, Matrouh University, Matrouh, Egypt

**Keywords:** Telmisartan, Fipronil, MNF2, DRP1, NRF2

## Abstract

Fipronil (FIP), as a broad-spectrum pesticide, contributes to adverse reproductive effects. Thus, our study aimed to investigate the effect of telmisartan (TEL) as a selective angiotensin 1 receptor (AT1R) blocker on mitochondrial damage induced by FIP intoxication in male rats. Forty healthy male albino rats were allocated into 4 groups (10/ group): the control group, the TEL group received TEL (10 mg/kg b.wt.), the FIP group received FIP (1/10 of the LD50 of 97 mg/kg b.wt.), and the FIP + TEL cotreated group. All treatments were taken orally for 60 days. Before the experiment, *in-silico* assessments of FIP and TEL were done. FIP administration diminished relative testicular weight, sperm count, and motility, besides significantly increasing sperm abnormalities (*p* < 0.05). Biochemically, FIP treatment reduced considerably (*p* < *0.05*) serum testosterone, luteinizing hormone (LH), and follicular-stimulating hormone (FSH) levels related to control animals. Furthermore, FIP administration significantly increased testicular malondialdehyde (MDA), pro-inflammatory markers as tumor necrosis factor-*α* (TNF-*α*), and interleukin-1*β* (IL-1*β*) levels. Also, downregulated the activities of antioxidant markers, Nrf2, HO-1, and PCNA immunostaining. Furthermore, FIP significantly upregulated (*p* < 0.05) DRP1 and downregulated PGC-1*α*, MNF2, TFAM, and mtDNA mRNA transcripts. Histopathologically, FIP induced deterioration in seminiferous tubules' histoarchitecture with upregulation in Cosentino's score and downregulation in Johnson's score. On the contrary, TEL effectively restored the testicular function hormones, testicular and epididymal histoarchitecture, antioxidant indices, PGC-1*α*, and TFAM, with downregulation in MDA levels and DRP1 mRNA transcript. In conclusion, TEL protects the testicular mitochondria against damage from FIP toxicosis by modulating the Nrf2/HO-1/PGC-1α/MNF2/DRP1 expressions.

**Graphical Abstract:**

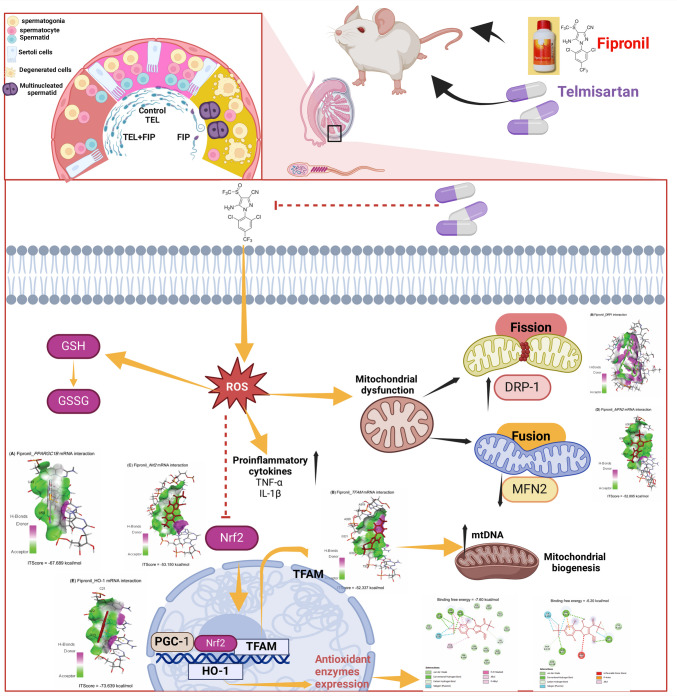

## Introduction

Fipronil (FIP) is an N-phenylpyrazole insecticide with numerous applications in veterinary medicine and agriculture. The World Health Organization (WHO) has classed FIP or 5-amino-3-cyano-1-(2,6-dichloro-4-trifluoromethylphenyl)−4-fluoromethylsulfinyl pyrazole as a class II moderately toxic pesticide (Chagnon et al. 2015). FIP desulfinyl, a primary breakdown product, is typically more hazardous than the parent molecule and has significant persistence. It causes severely detrimental effects on aquatic organisms and upland birds, and is moderately harmful to mice and rats, while proving non-toxic to waterfowl and other avian species (Kitulagodage 2011). The acute oral LD50 is 97 mg/kg, but the cutaneous LD50 exceeds 2000 mg/kg in rats (Gaines 1969). FIP was classified as a group C carcinogen because of the elevated incidence of thyroid follicular cell tumors in both male and female rats (World Health Organization 2020). FIP disturbs the central nervous system of insects by blocking Cl-channels linked to gamma-aminobutyric acid (GABA) receptors, resulting in the death of insects by neuronal paralysis and hyperexcitation (Bae and Kwon 2020). Moreover, FIP enhances DNA damage and apoptosis in rat spermatozoa, leading to adverse effects on male fertility (Bae and Kwon 2024; Khan et al. 2015). The prior work indicated that FIP binds with GABAA receptors, displacing GABA, which could serve as a significant regulator of sperm (Bae and Kwon 2020). Mazzo et al. (2018) found that male rats given FIP at 5 mg/kg for 14 days had lower sperm production, epididymal sperm count, glutathione (GSH) levels, and increased malondialdehyde (MDA) concentrations. Despite these potential toxicological effects, research into the molecular mechanism of FIP's influence on sperm function remains in its early phases.

The testicular renin-angiotensin system (RAS) is responsible for maintaining the levels of seminal electrolytes, regulating steroidogenesis and spermatogenesis, and sperm functionality (Gianzo and Subirán 2020). On the other hand, angiotensin II is the main component of the RAS and through its binding to angiotensin receptor (AT1R) decreases testosterone levels (Leung et al. 2000) and causes tissue oxidative stress, apoptosis (Eid et al. 2016), and inflammation by upregulating the proinflammatory transcription factors, resulting in a wide range of pro-inflammatory cytokines that further harm the tissues (Hayashi et al. 2010). Inhibition of angiotensin-converting enzyme or direct blocking of AT1R are the two main ways to inhibit the RAS (Imenshahidi et al. 2024). Angiotensin receptor blockers (ARBs) are recognized as safe and effective antihypertensive agents that mitigate the adverse effects of angiotensin II, a hormone implicated in fibrosis and cardiac failure, at AT1R (Galzerano et al. 2010; Hasegawa et al. 2011). Telmisartan (TEL) has the highest binding affinity among all commercially available ARBs for the AT1R (Kushwaha and Jena 2013). TEL exerts its antioxidant and anti-inflammatory actions via two metabolic pathways: activation of peroxisome proliferator-activated receptor-*γ* (PPAR-*γ*) and blockade of AT1R (Almukainzi et al. 2024; Destro et al. 2011). Also, it has the most extended half-life compared to the other ARBs (Ayza et al. 2020). TEL's elimination half-life is around 24 h, resulting in a long-lasting action and sustained blood pressure decreases (Sharpe et al. 2001). Additionally, it has been shown that TEL can preserve the mitochondrial functional activity via activation of the AKT/Glycogen synthase kinase-3 beta (GSK3β)/Peroxisome proliferator-activated receptor-*γ* coactivator 1-*α* (PGC1*α*) pathway in Parkinson’s model (Ray et al. 2024). In testicular tissues, it was found that TEL significantly inhibited the arsenic-induced expression of caspase-3 and had a significant anti-apoptotic effect in diabetic rat germ cells by inhibiting caspase-3 activity (Fouad et al. 2015). Additionally, it was shown that TEL can reduce cadmium-induced testicular damage through pathways involving the inducible nitric oxide synthase pathway, nuclear factor-kappa B (NF-*κ*B), Fas ligand, and caspase-3 (Dabbs 2021). To our knowledge, the preventive effect of TEL (AT1-receptor blocker) against testicular damage caused by FIP toxicity has not been previously reported.

Mitochondria are dynamic organelles highly susceptible to degeneration from oxidative damage (Kubli and Gustafsson 2012). The mitochondrial dynamics (fission and fusion) are balanced inside cells. The mitochondrial fusion process is facilitated by the GTPase proteins mitofusin 1 (MFN1) and mitofusin 2 (MFN2), which are situated on the outer mitochondrial membrane (Chen et al. 2011). Mitochondrial fusion is regulated in conjunction with mitochondrial fission, which is partially facilitated by the cytosolic protein dynamin-related protein 1 (DRP1) and its associated protein, mitochondrial fission 1 (Yoon et al. 2003). Mitochondrial biogenesis is a meticulously regulated process governed by nuclear transcription factors, including nuclear respiratory factor-1 and its coactivator peroxisome proliferator-activated receptor*γ* coactivator 11*β* (PPARGC1*β*), which enhance the expression of mitochondrial transcription factor A (TFAM), thereby facilitating the replication of mitochondrial DNA (mtDNA) (Andersson and Scarpulla 2001; Hull et al. 2016). Abnormal or depolarized mitochondria, which are significant sources of ROS formation, undergo fission and are eliminated from cells by mitophagy (Frank et al. 2012; Green and Kroemer 1998). The effect of peroxisome proliferator-activated receptor gamma coactivator-1*α* (PGC-1*α*), nuclear factor erythroid 2-related factor-2 (Nrf2), and heme oxygenase-1 (HO-1) on mitochondrial dynamics in testicular tissue remains inadequately understood.

Thus, this investigation aimed to assess the preventive effect of TEL in rats suffering from FIP-induced testicular damage and dysfunction. The potential mechanisms underlying these protective benefits were investigated by shedding light on their role against oxidative stress (Nrf2, HO-1, and glutathione system) and mitochondrial dynamics (PGC-1*α*, MFN2, DRP1, TFAM, and mtDNA).

## Materials and Methods

### Ethical statement

The Institutional Animal Care and Use Committee (IACUC), Alexandria University, Egypt, authorized the experimental procedures under approval number (ALEXU- IACUC, 013–2024-12–09/330). All methods adhered to the ARRIVE standards (Percie du Sert et al. 2020). Careful measures were taken to reduce the number of animals used and to alleviate their pain, including careful handling to prevent undue disruptions, compression, pressure, or painful manipulation.

### Chemicals and reagents

FIP was obtained as a marketable product named Fipro-Force (Mobidat Co, Egypt) with a concentration of 200 g/L. TEL was acquired as a saleable product, Mycardis 80 mg (*Boehringer Ingelheim*). Biochemical kits were purchased from Biodiagnostic Co. (Cairo, Egypt) and Abcam (Cambridge, United Kingdom). All other chemicals used in the study were of the highest commercially available analytical grade and purity.

### Molecular docking assessment

The three-dimensional (3D) structures of rats’ catalase (CAT), glutathione peroxidase-1 (GPx-1), PPARGC1*β*, TFAM, Nrf2, MFN2, HO-1, and DRP1 proteins were retrieved from the UniProt database (https://www.uniprot.org/). Besides, the mRNA sequences of rats’ PPARG coactivator-1*β* (*PPARGC1B*), *TFAM*, *Nrf2*, *MFN2*, and *HMOX1* were sourced from the NCBI-nucleotide database (https://www.ncbi.nlm.nih.gov/nucleotide/) and used for their 3D structures using BIOVIA Discovery Studio software. Regarding ligands, the 3D structures of FIP and TEL were retrieved from the PubChem database (https://pubchem.ncbi.nlm.nih.gov/).

The molecular docking interactions of FIP and TEL with mRNAs were done using NLDock_v1.0 with Ubuntu 22.04.2 LTS and finally visualized by the Discovery Studio 2016 Client software. The molecular docking interactions of FIP and TEL with protein receptors were done using Chimera 1.16 with AutoDock Vina and visualized using Discovery Studio 2016 Client software.

### Experimental design

Forty male albino rats that appeared to be in good health, weighing between 200–220 g at 8 weeks, were used in the experiment. Rats were acquired from the Medical Research Institute, Alexandria University, and kept in the laboratory of the Pharmacology Department, Faculty of Veterinary Medicine, Alexandria University. Rats were accommodated in well-ventilated polycarbonate cages with sawdust bedding, with 10 rats per cage, under a natural dark–light cycle at 21 ± 2 °C and 60% ± 2% humidity. The bedding is replaced every day to ensure a clean and dry environment. The rats were free from specific pathogens with unlimited access to a conventional diet and water. Before starting the experiment, the rats were adapted for two weeks.

Rats were randomly assigned to four distinct groups, each consisting of ten rats (n = 10), following these treatment protocols: Control group: Rats were administered 1 ml of saline solution; TEL group: Rats received TEL (10 mg/kg) (Wienen et al. 2001); FIP group: rats were administered the oral dose of FIP (1/10 of LD50 of 9.7 mg/kg) (Tomlin 2003); and the TEL + FIP group: Rats were given TEL and, one hour later, FIP at the same doses mentioned to avoid potential drug interaction for 60 days. All treatments were given orally and daily for 60 days, starting from the first day of the study.

### Sampling

At the end of the trial period, retro-orbital blood was collected in plain tubes under light isoflurane anesthesia with heparinized capillary tubes placed into the rat's medial canthus of the eye. Then, blood was allowed to coagulate for 30 min at 25 degrees and centrifuged for 15 min. After that, the serum layer was separated into a clean Eppendorf tube for further biochemical analysis. Then, the rats were euthanized, and their testicles with epididymis were carefully dissected. The right one was fixed for histopathological and immunohistochemical analysis in 10% neutral buffered formalin. At the same time, the left testicles were rapidly excised and washed in saline for homogenization. One gram of testicular tissue was homogenized in phosphate buffer saline (PBS) at pH 7.4. Then, the testicular homogenate was centrifuged at 1200 × g for 20 min at 4 °C, and the supernatants were separated and preserved at − 80 °C for further biochemical and gene expression analysis.

### Testicular and epididymal weights

Each rat's testis and epididymis were removed and weighed. The organ weight was estimated as the index weight (IW) = (organ weight (g)/body weight (g)) × 100, as previously documented by Matousek (1969).

### Epididymal sperm count, motility, and abnormality

Progressive sperm motility was estimated following Bearden and Fuquay (Bearden and Fuquay 1981). A drop of recently undiluted epididymal content was mixed with one drop of physiological saline on the warm slide and examined using the microscope's low-power objective lens (X10). For sperm abnormality evaluation, one drop of physiological saline was combined with one drop of each rat's epididymal contents and mixed with an eosin–nigrosin stain drop. The thin blood film was distributed across sanitized slides kept at room temperature to dry. Two hundred sperm were inspected on each slide under a high-power lens of the light microscope (X40), and ratios of nonstandard sperm (abnormalities in head and tail) were documented following Ikpeme et al. (2007)

For counting the number of epididymal sperm, a hemocytometer and a pipette used for RBCs counting were utilized. A drop of diluted cauda epididymal content was withdrawn up to the mark 0.5, and the pipette was then filled with a solution of sodium bicarbonate 5% and formalin up to the mark of 101. A few drops of fluid were expelled, and then a small amount of the diluted sperm suspension was located at the edge of the cover slide of the hemocytometer (Bearden and Fuquay 1981).

### Serum testosterone, FSH, and LH levels

Testosterone level was analyzed quantitively in serum using specific ELISA kits (Cat. No CSB-E05100r; CUSABIO Co.) according to Tietz (1995). Also, quantitative measurements of follicular-stimulating hormone (FSH) and luteinizing hormone (LH) were made in serum using specific ELISA kits from CUSABIO Co. (Cat. No CSB-E069r and CSB-E12654r, respectively). Following the manufacturer's guidelines, an ELISA microplate reader was used at 450 nm.

### Testicular oxidative and antioxidant biomarker assessment

Testicular MDA was quantified by the method of Draper and Hadley (1990). Total glutathione peroxidase (tGPx) and catalase (CAT) activities were assessed according to the techniques outlined by Flohé and Günzler (1984) and Beers and Sizer (1952), respectively. While total (tGSH), reduced (GSH), and oxidized glutathione (GSSG) contents were estimated based on the methodology of Griffith (1980). All prior parameters were measured spectrophotometrically according to the manufacturer's directions using Bio-diagnostic Co. (Dokki, Giza, Egypt) commercial kits.

#### Testicular proinflammatory cytokines assessment

Tumor necrosis factor-*α* (TNF-*α*) was quantitively detected in testicular homogenate using a rat TNF-*α* ELISA kit from Chongqing Biospes Co., Ltd. with Cat. No. BEK1214. While interleukin-1*β* (IL-1*β*) was quantitively determined using a rat IL-1*β* ELISA kit from CUSABIO Co. (Cat. No CSB-E08055r). All trials were prepared according to the manufacturer's directions.

#### Histopathological assessment

Fixed testicular and epididymal samples were processed using the conventional paraffin embedding technique. Then, sections are sliced with a 4 µm thickness and ultimately undergo Hematoxylin and Eosin (H&E) staining procedures (Suvarna et al. 2018).

The extent of testicular injury and the efficiency of spermatogenesis were evaluated using Cosentino's grading system Cosentino et al. (1985) and the mean Johnsen testicular biopsy score (MJTBS) (Dang-Cong and Nguyen-Thanh 2022), respectively. Cosentino's grading system categorizes the testis into four distinct classifications, denoting the spectrum from normal testicular parenchyma to necrotic parenchyma. In JTBS, a score of 10 indicates normal spermatogenesis, 9 denotes the presence of numerous spermatozoa with disarray in the germinal epithelium, and 8 signifies a scarcity of spermatozoa. Conversely, from 7 to 2 scale indicates the cessation of maturation, while a score of 1 represents the total absence of cellular structure within the seminiferous tubules. The average scores of Cosentino and Johnsen were computed and illustrated using graphs. A semiquantitative assessment of epididymal injuries was conducted following the methodology of Gibson-Corley et al. (2013). Lesions (Vacuolation and hyperplasia) in ten chosen micrographs were randomly selected from each slide for each rat, and then the average was estimated. The scored parameters were evaluated using the following scoring scale: 0 equal to normal, 1 ≤ 25%, 2 = 26–50%, 3 = 51–75%, and 4 = 76–100%.

#### Immunohistochemistry assessment

The slides underwent a process of deparaffinization and rehydration, subsequently being immersed in a 10 mM sodium citrate buffer solution and subjected to microwave heating to facilitate antigen retrieval. The endogenous peroxidase enzyme underwent inactivation through incubation with 3% hydrogen peroxide in absolute methanol at a temperature of 4 °C for 30 min, after which washing with phosphate-buffered saline (PBS) was performed. The occurrence of nonspecific binding was effectively inhibited by employing a 10% normal blocking serum for 60 min at room temperature. The first antibodies were subsequently incubated overnight at 4 °C against proliferating cell nuclear antigen (PCNA) and interleukin 1*β* (IL-1*β*) (Santa Cruz Biotechnology, Dallas, TX, USA), rinsed with PBS, and then incubated for 60 min with a second antibody. The slices were subsequently treated for 30 min with streptavidin-peroxidase conjugate following rinsing with PBS. The streptavidin–biotin complex was subsequently subjected to a 3-min incubation with a 3, 3′-diaminobenzidine tetrahydrochloride-H_2_O_2_solution at a pH of 7.0. Following this process, the sections were thoroughly rinsed with distilled water and subsequently stained utilizing Mayer’s hematoxylin (Dabbs 2021). PCNA and IL-1*β*immune expression intensity was assessed in 10 randomly selected fields from 6 animals in each group using FIJI ImageJ software (NIH, USA) (Crowe and Yue 2019).

#### Gene expression analysis

After homogenizing approximately 100 mg of testicular tissues in liquid nitrogen, the homogenate was kept at −80 °C until RNA isolation. The RNeasy Mini Kit (Qiagen GmbH, Germany) was used to isolate total RNA following the directions provided by the manufacturer. Following the manufacturer's instructions, reverse transcription was done using the TOPscript™ RT DryMIX (dT18/dN6 plus) kit (Enzynomics Co Ltd, Korea, cat number RT220). For TOPscript™ Reverse Transcriptase to be as stable as possible after drying with reaction buffer, dNTP mixture, stabilizer, oligo dT primer, and random hexamer primer, TOPscript™ Reverse Transcriptase DryMIX (dT18/dN6plus) is designed. A highly thermostable genetically modified form of M-MLV RT, TOPscript™ reverse transcriptase, can manufacture cDNA at temperatures as high as 60 °C. This characteristic is essential when long RNA templates contain many secondary structures. Messenger RNA can be converted into cDNA greater than 20 kb using TOPscript™ Reverse Transcriptase. ViPrime PLUS Taq qPCR Green Master Mix I (Vivantis Technologies, Malaysia, cat. No. QLMM12) was used to perform relative quantification of PGC-1*α*, TFAM, NRF2, DRP1, MFN2, and HO-1 expressions. Table 1lists the PCR conditions and the primer sequences for each target and reference gene. The data was collected by Bio-Rad CFX Maestro version 2.3 (Bio-Rad, Inc., USA). By computing and normalizing the target's threshold cycles (Ct) values to those of 18 s rRNA using the ΔΔCt method, the relative expression of target genes was measured concerning the expression of the reference gene (18S rRNA) in the same sample (Livak and Schmittgen 2001).

**Table 1:** Primers used for the qRT-PCR amplification

Genes	Accession number	Primers’ sequences	
**18S rRNA (Reference gene)**	**NR_046237.2**	**F**	GTAACCCGTTGAACCCCATT
		**R**	CAAGCTTATGACCCGCACTT
**PGC-1*α***	**NM_031347.1**	**F**	GTGCAGCCAAGACTCTGTATGG
		**R**	GTCCAGGTCATTCACATCAAGTTC
**TFAM**	**NM_031326.2**	**F**	CCCACAGAGAACAGAAACAG
		**R**	CCCTGGAAGCTTTCAGATACG
**HO-1**	**NM_012580.2**	**F**	CGTGCAGAGAATTCTGAGTTC
		**R**	AGACGCTTTACGTAGTGCTG
**Nrf2**	**NM_031789.2**	**F**	CAAATCCCACCTTGAACACA
		**R**	CGACTGACTAATGGCAGCAG
**DRP1**	**NM_053655.3**	**F**	GATGCCATAGTTGAAGTGGTGAC
		**R**	CCACAAGCATCAGCAAAGTCTGG
**MFN2**	**NM_130894.4**	**F**	GCCAGCTTCCTTGAAGACAC
		**R**	GCAGAACTTTGTCCCAGAGC

#### Mitochondrial DNA assessment

A qPCR assay was applied to evaluate the mtDNA copy number by detecting the ratio of PCR amplicons of mitochondrial sequence to that of a single nuclear gene in experimental samples (Gowayed et al. 2020). In this study, after total genomic DNA isolation using DNeasy® Kit (Qiagen, Germany, catalog number: 69504) under the manufacturer's instructions, a specific primer pair for mtDNA and PGC-1*α* (Table 1) was used to apply equal cycles of PCR and determine the relative mtDNA signal to nuclear DNA signal. The nuclear target was used to calculate nuclear DNA (nDNA) and normalize the amount of mtDNA per cell. The content of mtDNA was determined by the Ct (mtDNA)/Ct (nDNA) ratio. The relative number of mtDNA was estimated relative to PGC-1*α* by calculating and normalizing.

#### Statistical analysis

The Kolmogorov–Smirnov test assessed data normality. Values were represented as mean ± SE, and group comparisons were performed via analysis of variance (ANOVA) followed by the Tukey–Kramer post hoc test. Nonparametric lesion scoring was analyzed using Kruskal–Wallis’s test, followed by Dunn’s test. Significance was applied at *p*< 0.05. All statistical analyses were conducted via Prism software (GraphPad® Software Inc., Version 10.3.1, San Diego, CA, USA) (Festing and Altman 2002).

## Results

### Molecular docking interactions

**Fig. 1:**
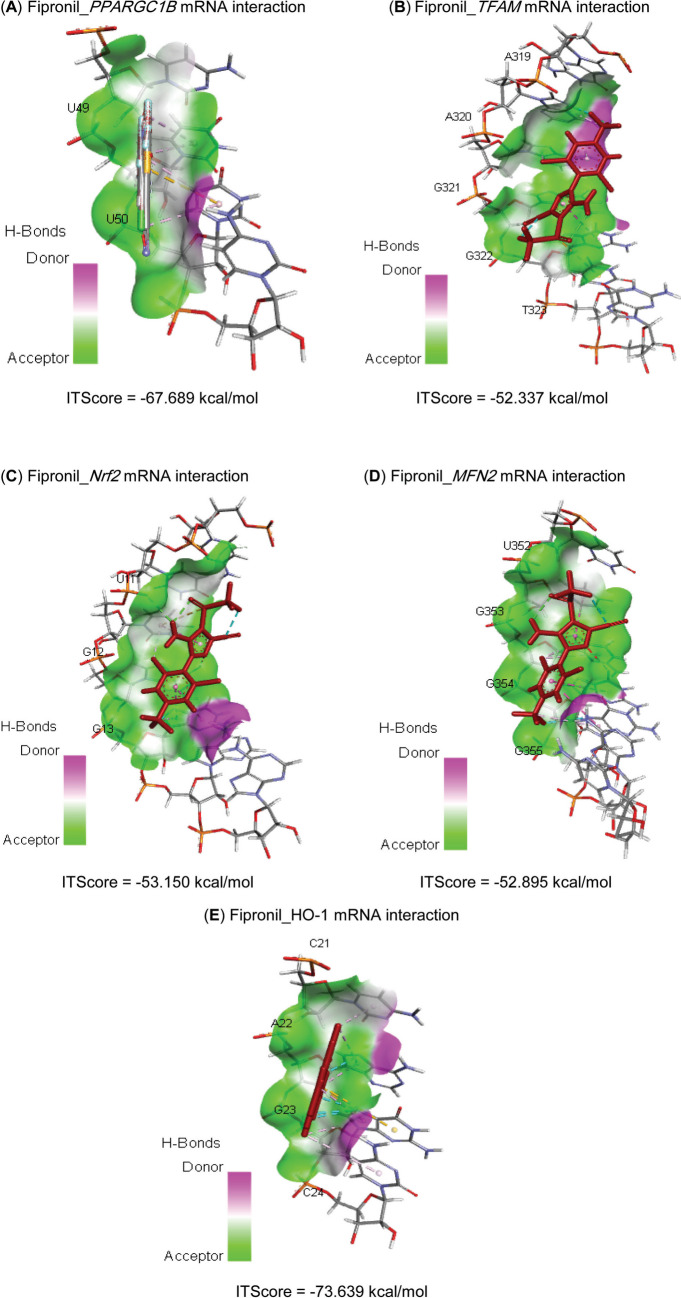
Molecular interaction of fipronil with rats’ (**A**) PPARG coactivator-1*β* (PPARGC1B), (**B**) mitochondrial transcription factor-A (TFAM), (**C**) nuclear factor erythroid 2-related factor-2 (Nrf2), (**D**) mitofusin-2 (MFN2), and (**E**) heme oxygenase-1 (HMOX1) mRNAs

**Fig. 2:**
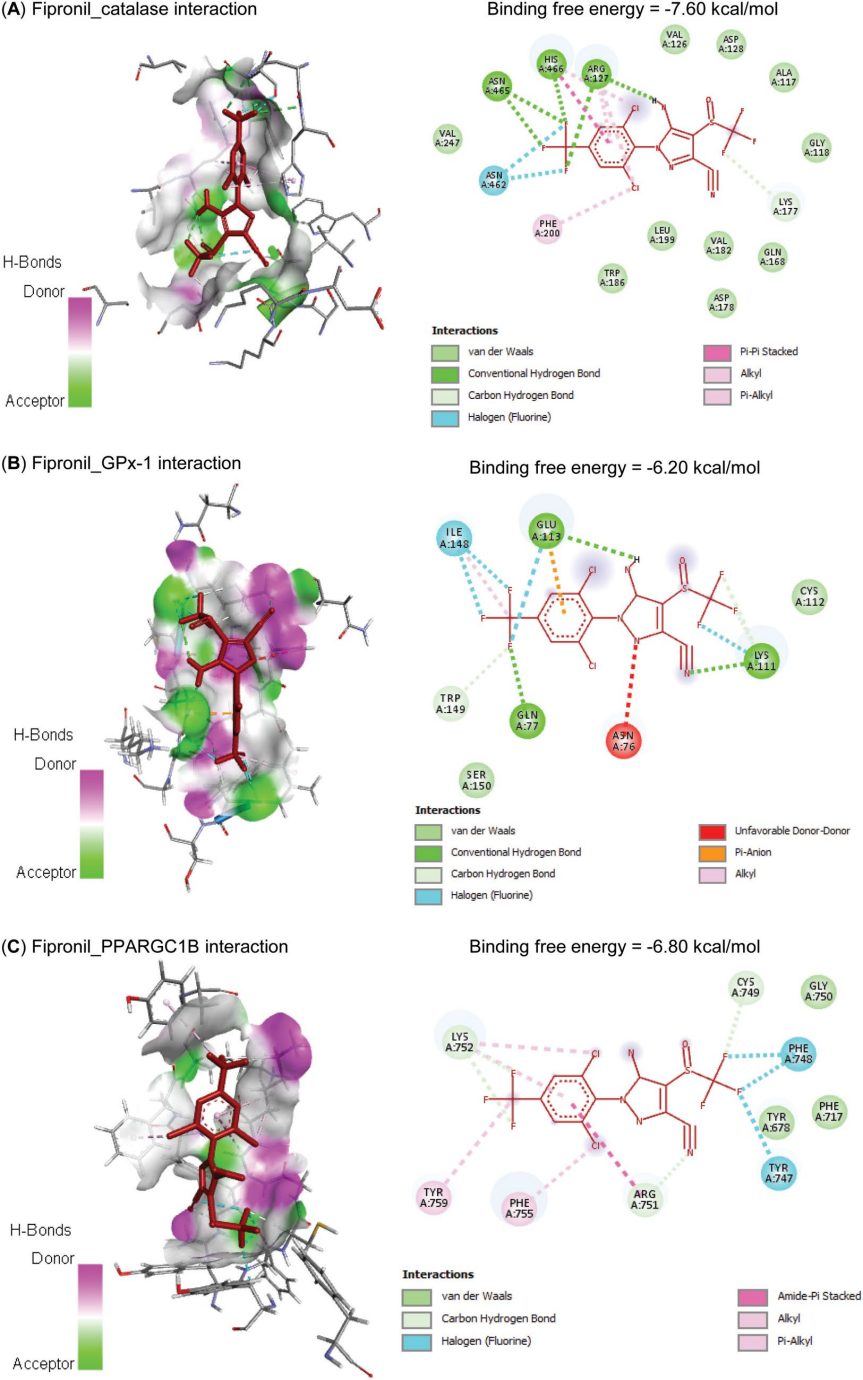

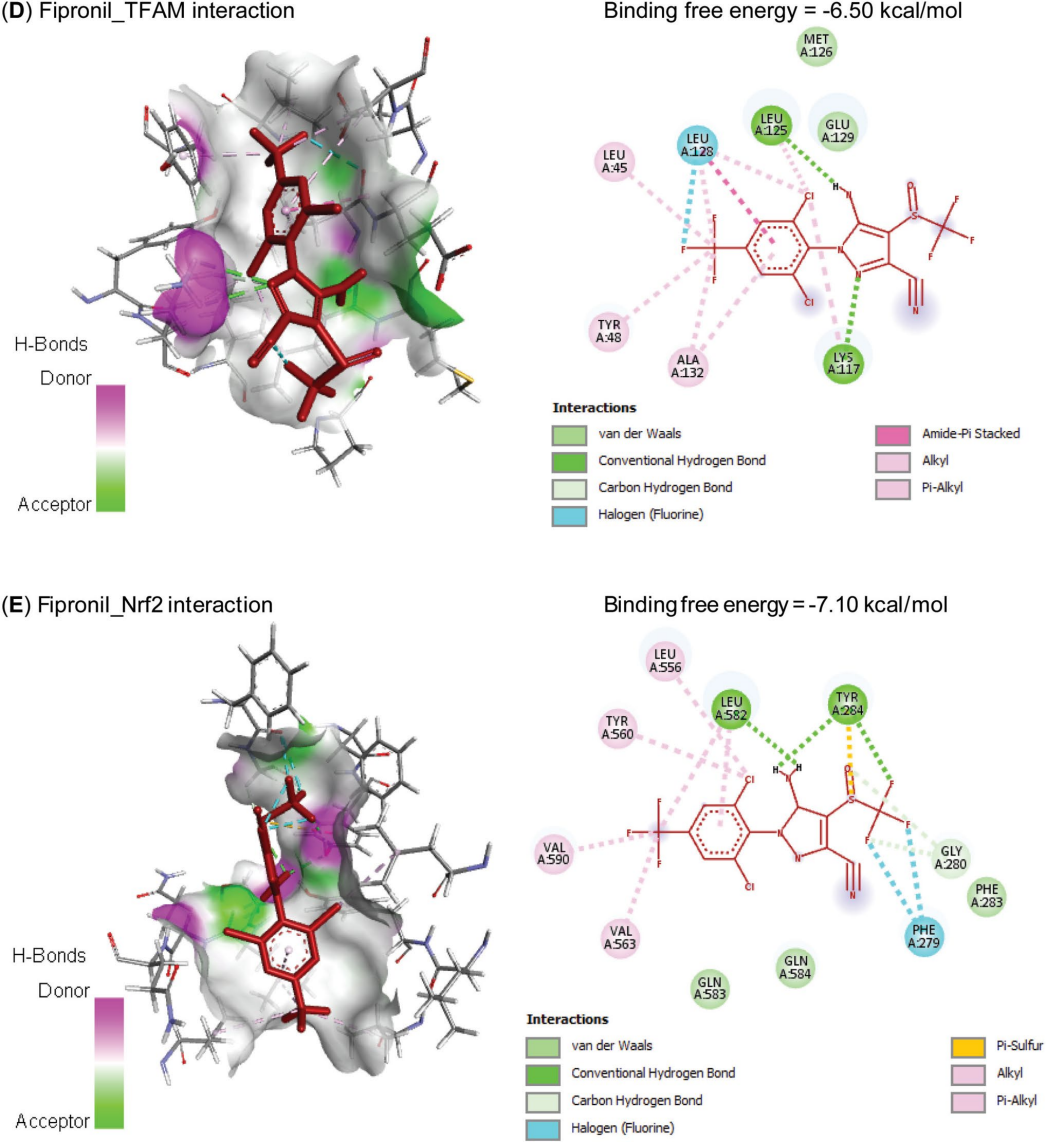

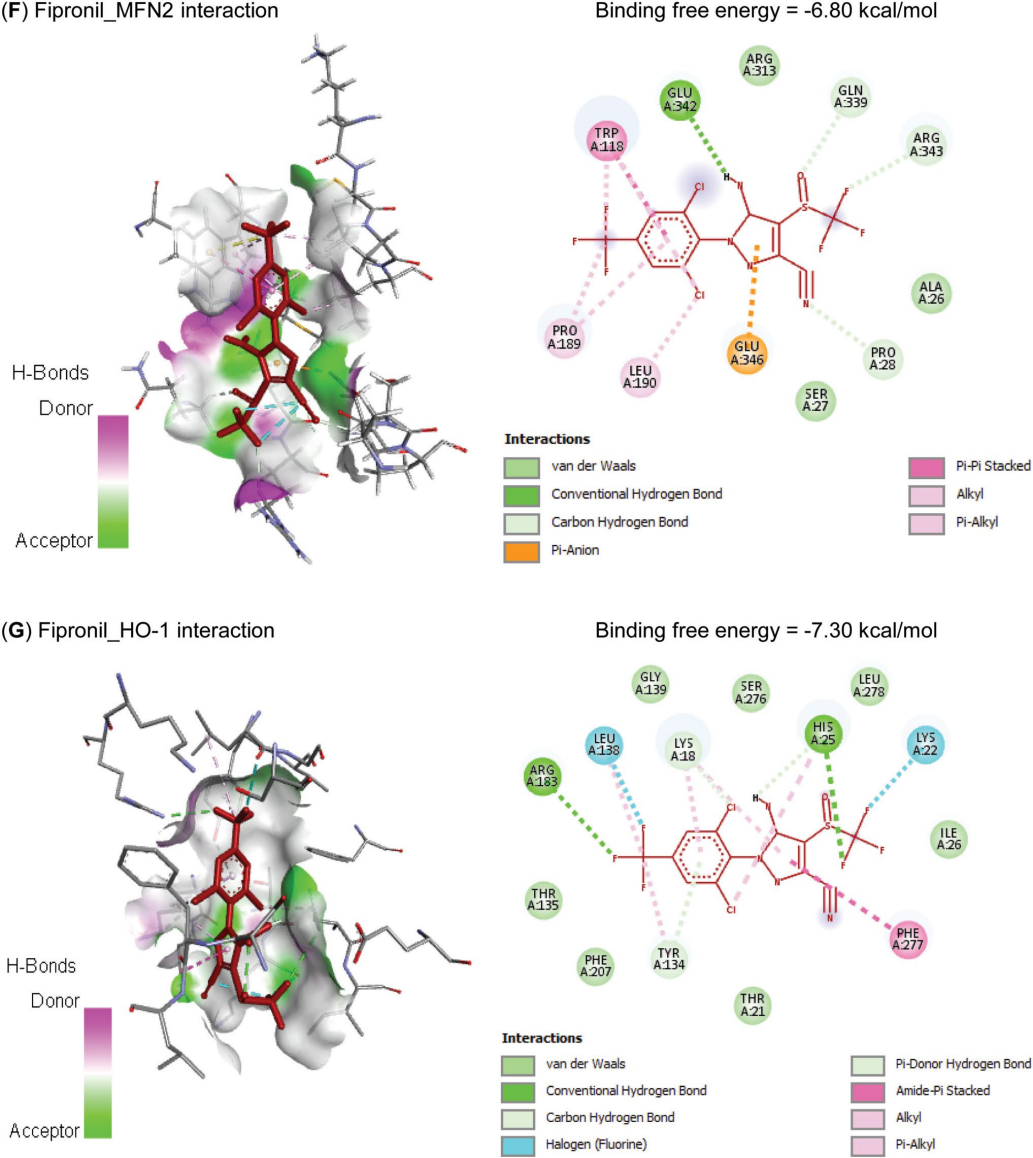
Molecular interaction of fipronil with rats’ (**A**) catalase (CAT), (**B**) glutathione peroxidase-1 (GPx-1), (**C**) PPARG coactivator-1*β* (PPARGC1B), (**D**) mitochondrial transcription factor-A (TFAM), (**E**) nuclear factor erythroid 2-related factor-2 (Nrf2), (**F**) mitofusin-2 (MFN2), and (**G**) heme oxygenase-1 (HO-1) proteins

Molecular interactions of FIP with rats’ PPARGC1*β*, TFAM, Nrf2, MFN2, and HMOX1 mRNAs are represented in Table 2 and Fig. 1 and exhibited binding free energies of −67.689, 52.337, −53.150, −52.895, and −73.639 kcal/mol, respectively. Also, FIP interacted with the binding sites of CAT (−7.60 kcal/mol), GPx-1 (−6.20 kcal/mol), PPARGC1*β* (−6.80 kcal/mol), TFAM (−6.50 kcal/mol), Nrf2 (−7.10 kcal/mol), MFN2 (−6.80 kcal/mol), and HO-1 (−7.30 kcal/mol) proteins (Table 3 and Fig. 2).

**Table 2:** Molecular interaction of fipronil with rats’ PPARG coactivator-1*β* (*PPARGC1B*), mitochondrial transcription factor-A (*TFAM*), nuclear factor erythroid 2-related factor-2 (*Nrf2*), mitofusin-2 (*MFN2*), and heme oxygenase-1 (*HMOX1*) mRNAs

Targets	Residues	Hydrogen bond	Charge	Hydrophobic interaction	Halogen	Other
***PPARGC1B***	U49	0	0	2	0	0
	U50	0	0	2	0	2
***TFAM***	A319	1	0	1	1	0
	A320	1	0	1	0	0
	G321	1	0	0	0	0
	G322	0	0	1	0	0
	T323	0	0	0	0	1
***Nrf2***	U11	1	0	1	0	1
	G12	0	0	1	0	0
	G13	0	0	1	1	0
***MFN2***	U352	0	0	1	0	0
	G353	0	0	1	0	1
	G354	0	0	1	0	0
	G355	0	0	1	1	0
***HMOX1***	C21	0	0	1	0	0
	A22	0	0	1	1	0
	G23	0	0	1	1	1
	C24	0	0	1	0	0

**Table 3:** Molecular interaction of fipronil with rats’ catalase (CAT), glutathione peroxidase-1 (GPx-1), PPARG coactivator-1*β* (PPARGC1B), mitochondrial transcription factor-A (TFAM), nuclear factor erythroid 2-related factor-2 (Nrf2), mitofusin-2 (MFN2), and heme oxygenase-1 (HO-1) proteins

Targets	Residues	Hydrogen bond	Charge	Hydrophobic interaction	Halogen	Other
**CAT**	ARG127	1	0	1	1	0
	LYS177	1	0	0	0	0
	PHE200	0	0	1	0	0
	ASN462	0	0	0	1	0
	ASN465	1	0	0	1	0
	HIS466	1	0	1	1	0
**GPx-1**	GLN77	1	0	0	1	0
	LYS111	1	0	0	1	0
	GLU113	1	1	0	1	0
	ILE148	0	0	1	1	0
	TRP149	1	0	0	1	0
**PPARGC1B**	TYR747	0	0	0	1	0
	PHE748	0	0	0	1	0
	CYS749	1	0	0	1	0
	ARG751	1	0	1	0	0
	LYS752	1	0	1	0	0
	PHE755	0	0	1	0	0
	TYR759	0	0	1	0	0
**TFAM**	LEU45	0	0	1	0	0
	TYR48	0	0	1	0	0
	LYS117	1	0	1	0	0
	LEU125	1	0	1	0	0
	LEU128	0	0	1	1	0
	ALA132	0	0	1	0	0
**Nrf2**	PHE279	0	0	0	1	0
	GLY280	1	0	0	1	0
	TYR284	1	0	0	1	1
	LEU556	0	0	1	0	0
	TYR560	0	0	1	0	0
	VAL563	0	0	1	0	0
	LEU582	1	0	1	0	0
	VAL590	0	0	1	0	0
**MFN2**	PRO28	1	0	0	0	0
	TRP118	0	0	1	0	0
	PRO189	0	0	1	0	0
	LEU190	0	0	1	0	0
	GLN339	1	0	0	0	0
	GLU342	1	0	0	0	0
	ARG343	1	0	0	0	0
**HO-1**	LYS18	1	0	1	0	0
	LYS22	0	0	0	1	0
	HIS25	1	0	1	1	0
	TYR134	1	0	1	0	0
	LEU138	0	0	1	1	0
	ARG183	1	0	0	1	0

**Fig. 3:**
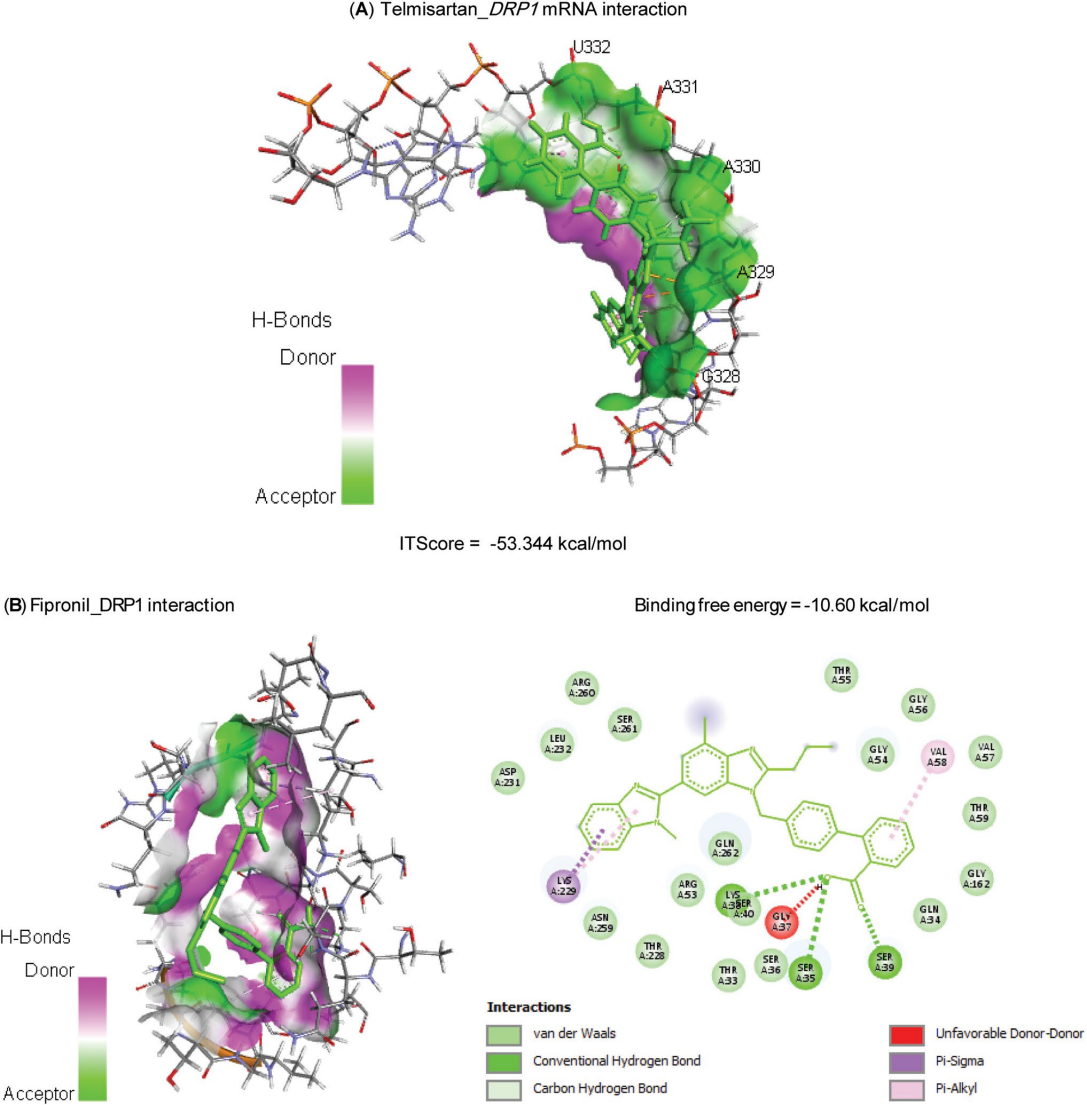
Molecular interaction of telmisartan with rats’ (**A**) dynamin-related protein-1 (DRP1) mRNA and (**B**) protein

Data in Table 4 and Fig. 3 explored the molecular interactions of TEL with rats’ DRP1 mRNA and protein, with binding free energies of −53.344 and −10.60 kcal/mol, respectively.

**Table 4:** Molecular interaction of telmisartan with rats’ dynamin-related protein-1 (DRP1) mRNA and protein

Targets	Residues	Hydrogen bond	Charge	Hydrophobic interaction	Halogen	Other
**mRNA**	G328	1	0	1	0	0
	A329	1	1	1	0	0
	A330	1	0	0	0	0
	A331	1	0	1	0	0
	U332	1	0	1	0	0
**Protein**	SER35	1	0	0	0	0
	LYS38	1	0	0	0	0
	SER39	1	0	0	0	0
	VAL58	0	0	1	0	0
	LYS229	0	0	1	0	0

### Effect of telmisartan on the reproductive organs’ weight changes induced by fipronil

Testicular weights and indexes in the FIP-treated group significantly declined compared to the control group. The relative index weight was preserved in the group's control values and simultaneously assigned with TEL + FIP (Table 5).

**Table 5:** Effects of telmisartan (TEL) on testis/body weight ratio in fipronil (FIP)-induced testicular damage in male albino rats

Experimental Group	Body weight (g)		Testicular weight (g)	Testicular index
	Initial	Final		
**Control**	174.8 ± 1.56	240.0 ± 13.04 ^a^	3.200 ± 0.31 ^a^	1.35 ± 0.16 ^a^
**TEL**	172.2 ± 1.88	232.0 ± 11.14 ^ab^	3.160 ± 0.17 ^a^	1.36 ± 0.03 ^a^
**FIP**	172.0 ± 1.00	158.8 ± 2.85 ^c^	1.380 ± 0.14 ^b^	0.87 ± 0.09 ^b^
**TEL + FIP**	171.4 ± 1.88	204.0 ± 2.21 ^b^	3.100 ± 0.05 ^a^	1.51 ± 0.01 ^a^

Values represented as mean ± SE (n = 5/group), means carrying different superscript letters are significantly different at *p*-value < 0.05

### Effect of telmisartan on sperm count, motility, and morphology alterations induced by fipronil

**Fig. 4:**
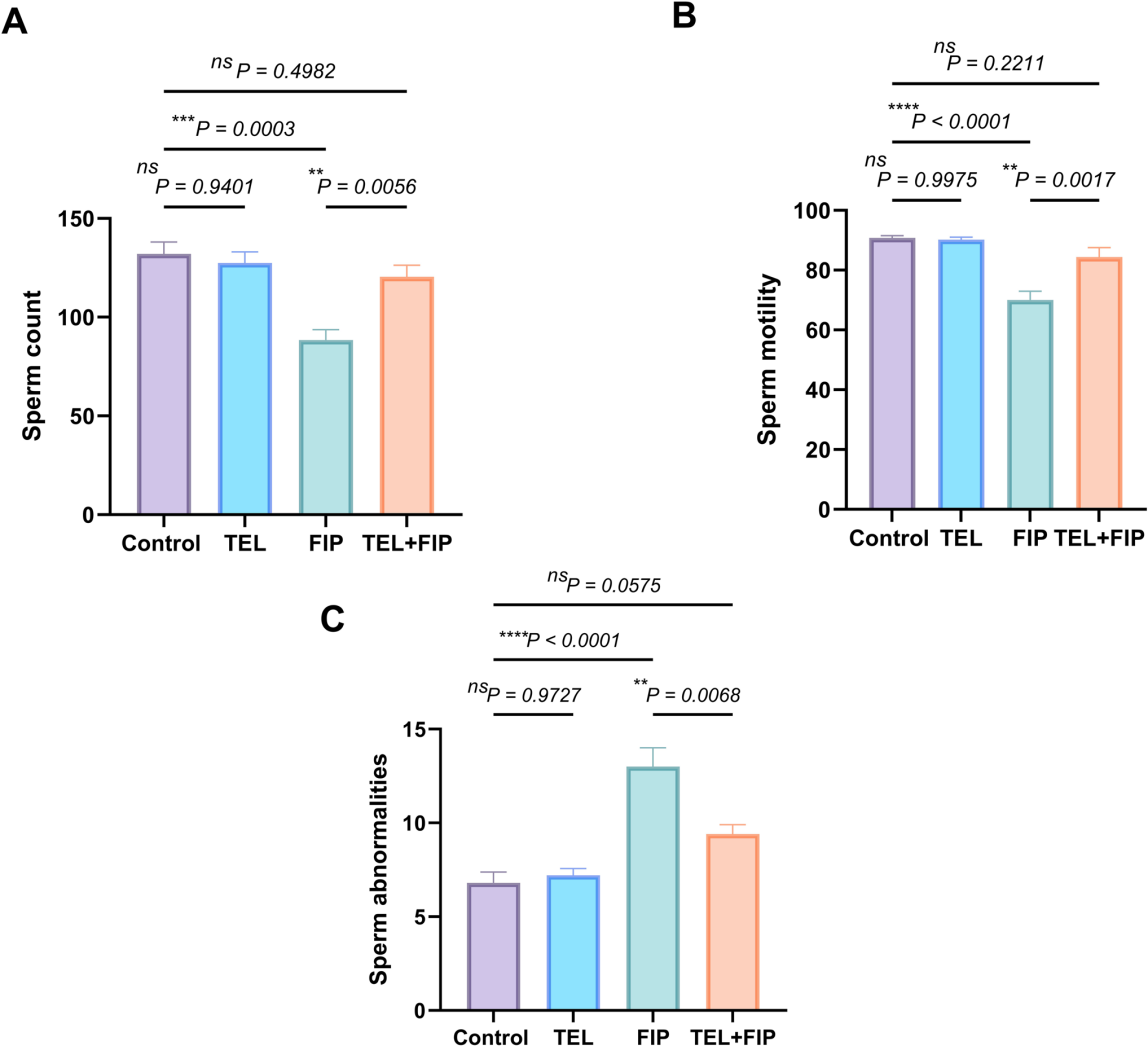
Effect of telmisartan (TEL) and fipronil (FIP) on: (**A**) sperm count, (**B**) sperm motility and (**C**) sperm abnormalities in male rats. Values presented as M ± SEM were assessed using a one-way ANOVA test, accompanied by Tukey’s post hoc test. *p < 0.05, **p < 0.01, ***p < 0.001, ****p < 0.0001

Figure 4 revealed that, in the FIP-treated group, there was a remarkable decline in sperm count and progressive sperm motility of 33 and 22.90%, respectively, compared to the control group. Conversely, rats treated with FIP concomitantly with TEL showed an increase in sperm count and progressive sperm motility by 24.24 and 20.5%, respectively, compared to the FIP-treated group. However, there was a substantial upsurge in the percentage of sperm abnormality in rats treated with FIP by 91.7% compared to the control. However, a remarkable reduction in sperm abnormalities (7.6%) was shown in the TEL + FIP co-treated group compared to the FIP-treated one. However, there were irrelevant changes in all sperm characteristics in TEL-treated rats as matched with the control rats.

### Effect of telmisartan on serum testosterone, FSH, and LH level alterations induced by fipronil

**Fig. 5:**
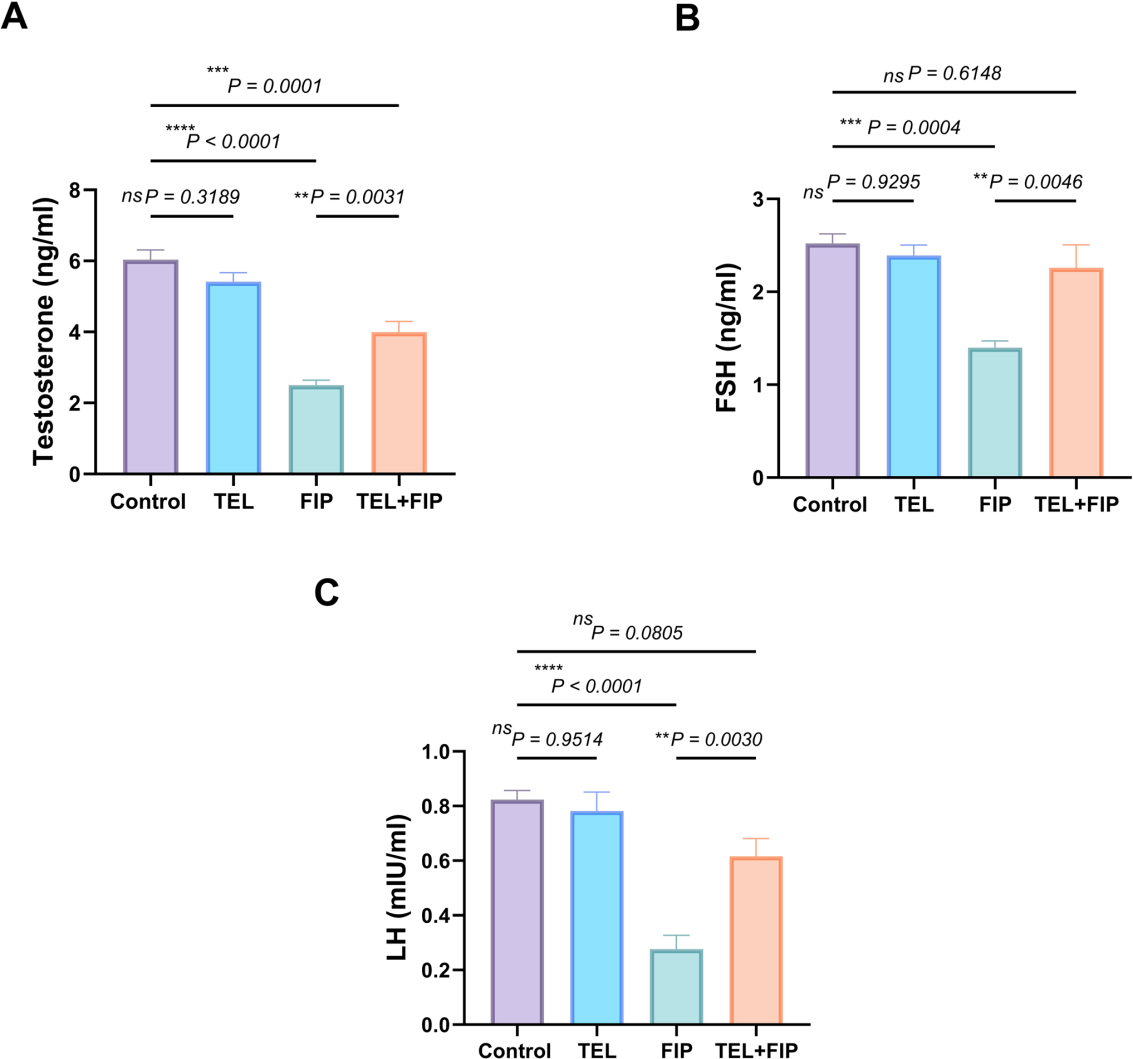
Effect of telmisartan (TEL) and fipronil (FIP) on serum levels of (**A**) testosterone, (**B**) FSH (follicular stimulating hormone), and (**C**) LH (luteinizing hormone) in male rats. Values presented as M ± SEM were assessed using a one-way ANOVA test, accompanied by Tukey’s post hoc test. *p < 0.05, **p < 0.01, ***p < 0.001, ****p < 0.0001

As shown in Fig. 5, sex hormones (testosterone, LH, and FSH) did not show any noteworthy changes between control and TEL-treated groups. In contrast, FIP-intoxicated rats revealed a substantial decline in serum testosterone, FSH, and LH levels (58.5, 44.5, and 66.5%, respectively) compared to the control group. Conversely, the TEL + FIP co-treated group showed significant increments in serum testosterone, FSH, and LH levels by 59.4%, 61.5%, and 123.2%, respectively, compared to FIP-intoxicated rats.

### Effect of telmisartan on the testicular oxidative/antioxidant biomarkers alterations induced by fipronil

**Fig. 6:**
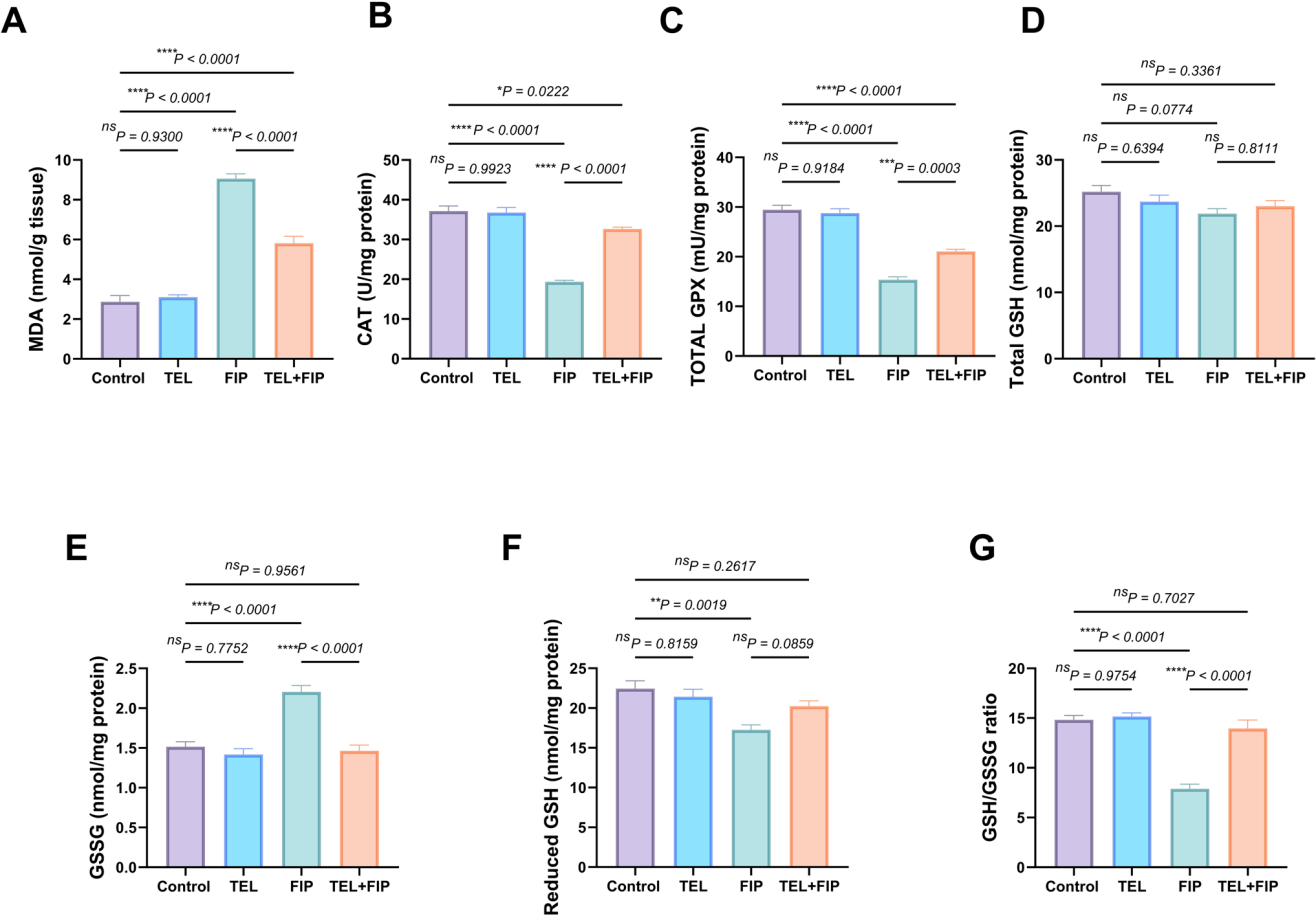
Effect of telmisartan (TEL) and fipronil (FIP) on oxidative/antioxidant biomarkers: (**A**) Malondialdehyde (MDA), (**B**) catalase (CAT), (**C**) Total glutathione peroxidase (tGPX), (**D**) Total glutathione (tGSH), (**E**) oxidized glutathione (GSSG), (**F**) reduced glutathion (GSH) and (**G**) GSH/GSSG in male rats. Values presented as M ± SEM were assessed using a one-way ANOVA test, followed by Tukey’s post hoc test. *p < 0.05, **p < 0.01, ***p < 0.001, ****p < 0.0001

FIP intoxication significantly increased the testicular MDA level (217%), with a significant decline in the testicular CAT (47.9%) and tGPx (47.9%) activities compared to control rats. Conversely, the co-treatment with TEL significantly decreased MDA level (36%) with a significant increase in CAT (68.6%) and tGPx (37.16%) activities. Non-significant alterations were detected in the testicular tGSH between all treated groups and the control. Also, FIP-treated rats demonstrated a significant increase in GSSG (45.6%) with a considerable decline in GSH contents (23.07%) and GSH/GSSG ratio (46.8%) relative to the control group. The co-treatment with TEL restored the glutathione system to its normal status. Also, substantial downregulations in GSSG (33.6%) and upregulation in GSH and GSH/GSSG (17.1% and 77.1%, respectively) were recognized compared to the FIP-intoxicated rats (Fig. 6).

### Effect of telmisartan on the testicular TNF-α and IL-1β levels alterations induced by fipronil

**Fig. 7:**
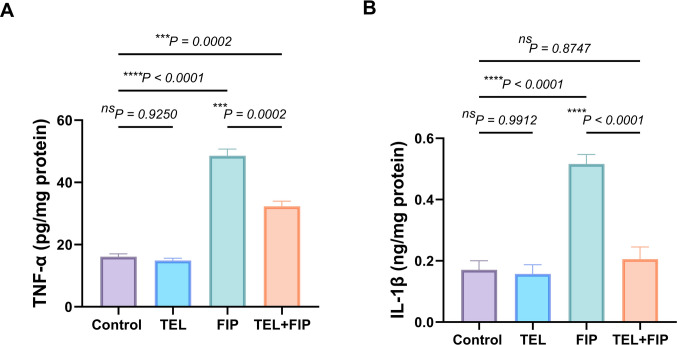
Effect of telmisartan (TEL) and fipronil (FIP) on testicular levels of (**A**) tumor necrosis factor-*α* (TNF-*α*) and (**B**) interleukin-1*β* (IL-1*β*) in male rats. Values presented as M ± SEM were assessed using a one-way ANOVA test, followed by Tukey’s post hoc test. *p < 0.05, **p < 0.01, ***p < 0.001, ****p < 0.0001

Regarding proinflammatory cytokines, there were no noteworthy changes in the testicular TNF-*α* and IL-1*β* levels between control and TEL-treated rats. However, they were substantially upregulated by 201% and 202.34%, respectively, in FIP-intoxicated rats compared to control animals. However, compared to FIP-intoxicated rats, the co-treatment with TEL decreased their levels by 33.4% and 60.2%, respectively (Fig. 7).

### Effect of telmisartan on the testicular and epididymal histopathological alterations induced by fipronil

**Fig. 8:**
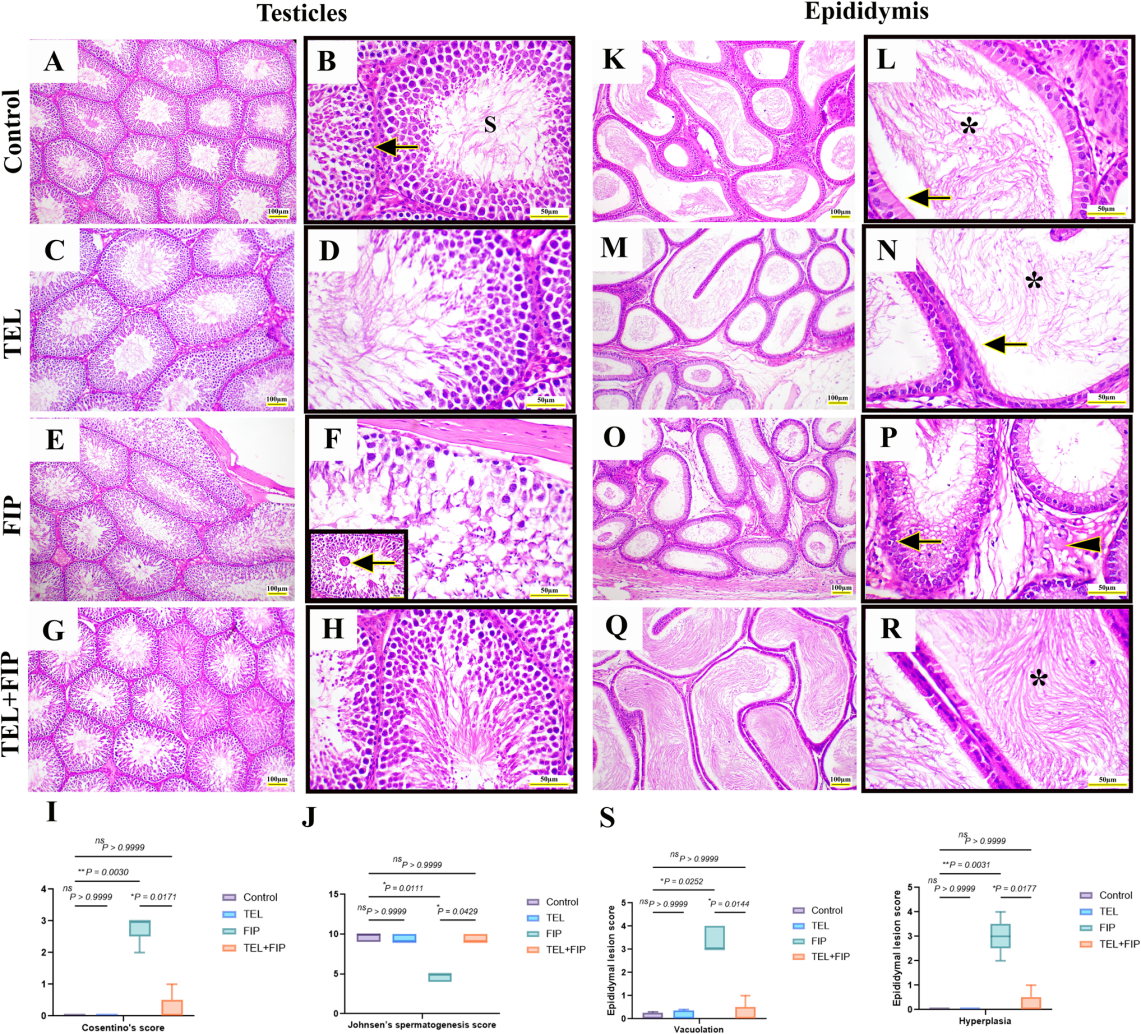
Effect of telmisartan (TEL) against fipronil (FIP) induced testicular and epididymal injury in rats. Hematoxylin and Eosin (H&E): (**A **& **B**) Testicular tissue of the control group showing normal spermatogenesis with typical spermatogonia (arrows) and numerous intraluminal spermatozoa (S), (**C** & **D**) Testicular tissue of TEL treated group, (**E** & **F**) Testicular tissue of FIP group showing degeneration and sloughing of germinal epithelium with formation of multinucleated giant cells (arrow), (**G** & **H**) Testicular tissue of TEL + FIP treated showing improvement of testicular histoarchitecture, (**I**) Testicular Cosentino’s lesion score. (**J**) Johnsen’s spermatogenesis score, (**K** & **L**) Epididymal tissue of control showing normal ciliated epithelium (arrow) with numerous intraluminal spermatozoa (*), (**M** & **N**) Epididymal tissue of TEL, (**O** & **P**) Epididymal tissue of the FIP group showing epithelial hyperplasia and vacuolation (arrow) and interstitial edema (arrowhead), (**Q** & **R**) Epididymal tissue of FIP group showing improvement of epididymal epithelium with numerous intraluminal spermatozoa and (**S**) Epididymal lesion score. Scale bar (A, C, E, G, K, M, O, Q) = 100 µm, Scale bar (B, D, F, H, L, N, P, R) = 50 µm and Scale bar (inset F) = 20 µm. Values presented as M ± SEM were assessed using a Kruskal–Wallis’s test followed by Dunn’s test. *p < 0.05, **p < 0.01, ***p < 0.001, ****p < 0.0001

The testicular tissues of control and TEL-treated animals exhibited typical interstitial tissue and normal-sized seminiferous tubules lined by spermatogenic cells with various stages of maturation. At the same time, the rat epididymis showed typical epithelium and luminal spermatozoa (Fig. 8A-D and K-N, respectively). On the other hand, FIP-treated rats exhibited degeneration and sloughing of spermatogenic cells with a decrease in intraluminal spermatozoa, besides the formation of multinucleated giant cells (Fig. 8E and F). Furthermore, the epididymal tissue of FIP-treated rats showed interstitial edema, vacuolations mediated by phospholipidosis, and epithelial hyperplasia (Fig. 8O and P). The testicles and epididymis of TEL + FIP-treated animals showed improved histoarchitecture with more dense spermatozoa in the lumen (Fig. 8G-H and Q-R). Semiquantitative statistical analysis of Cosentino's score in testicles exhibited a significant increase in degenerative changes compared to control and TEL-treated animals (Fig. 8I). However, the TEL + FIP group showed significant downregulation in the testicular lesions score relative to FIP-treated animals. Regarding Johnsen's score, FIP-treated animals showed considerable downregulation in Johnsen's score compared to the control and TEL rats. Furthermore, a significant upregulation in such scores was detected in the testicles of TEL + FIP-treated animals compared to FIP only (Fig. 8J). Epididymal lesion scores (Vacuolation and hyperplasia) significantly increased relative to control and TEL-treated rats. At the same time, TEL + FIP exhibited a significant downregulation of this score relative to the FIP group (Fig. 8S).

### Effect of telmisartan on PCNA and IL-1β immunoreactivity induced by fipronil

**Fig. 9:**
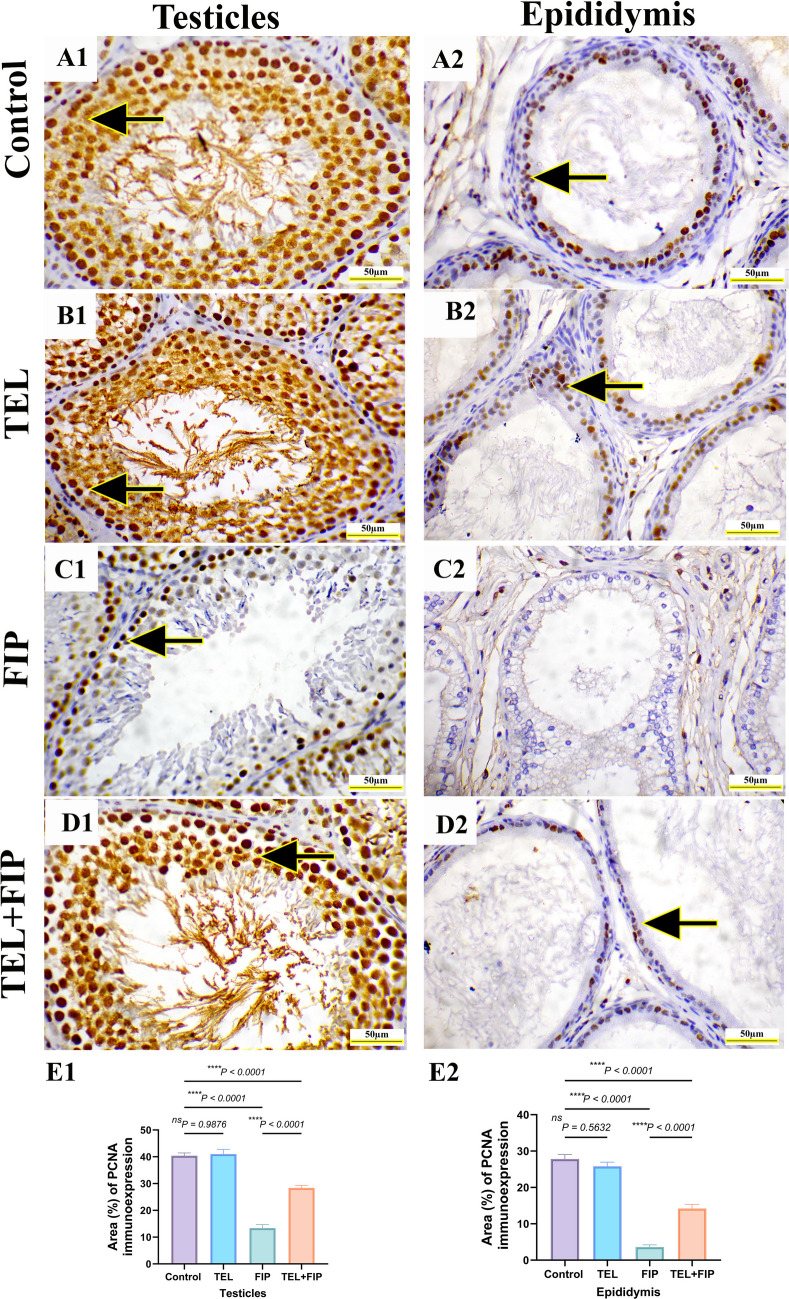
Effect of telmisartan (TEL) against fipronil (FIP) induced testicular and epididymal injury in rats. Immunohistochemical staining of proliferating cell nuclear antigen (PCNA). Scale bar = 50 µm. (**A1**&**A2**) Control group showing the intense PCNA immunoreaction in the nuclei of all spermatogenic cells and epididymal epithelium (arrows), (**B1**&**B2**) TEL-treated group. (**C1**&**C2**) FIP group showing low PCNA immunoreaction (arrows) in spermatogenic cells and epididymal epithelium, (**D1**&**D2**) TEL + FIP treated showing high PCNA immunoreactivity in spermatogenic cells and epididymal epithelium (arrows), and (**E1**&**E2**) Semi-quantitative analysis of PCNA area% in the testicular and epididymal tissues from various experimental groups. Values presented as M ± SEM were assessed using a one-way ANOVA test, followed by Tukey’s post hoc test. *p < 0.05, **p < 0.01, ***p < 0.001, ****p < 0.0001

PCNA immunohistochemical reactivity in rats’ testicles and epididymis revealed strong nuclear immunoreactivity in all spermatogenic cells and epididymal epithelium in control and TEL-treated animals (Fig. 9A1, B1, A2, and B2). Conversely, FIP-treated animals showed mild to nearly negative PCNA nuclear immunoreactivity in seminiferous tubules and epididymal epithelium (Fig. 9C1 and C2). TEL + FIP-treated animals revealed moderate to strong PCNA immunoreaction in the seminiferous tubules and epididymal epithelium (Fig. 9D1 and D2). Semi-quantification of PCNA area% in the testicles and epididymis exhibited a significant decrease in the immunoreactivity of PCNA in the FIP-treated animals relative to control and TEL-treated animals. This low immunoreactivity was substantially upregulated in the TEL + FIP-treated animals compared to FIP in both testicles and epididymis (Fig. 9E1 and E2).

**Fig. 10:**
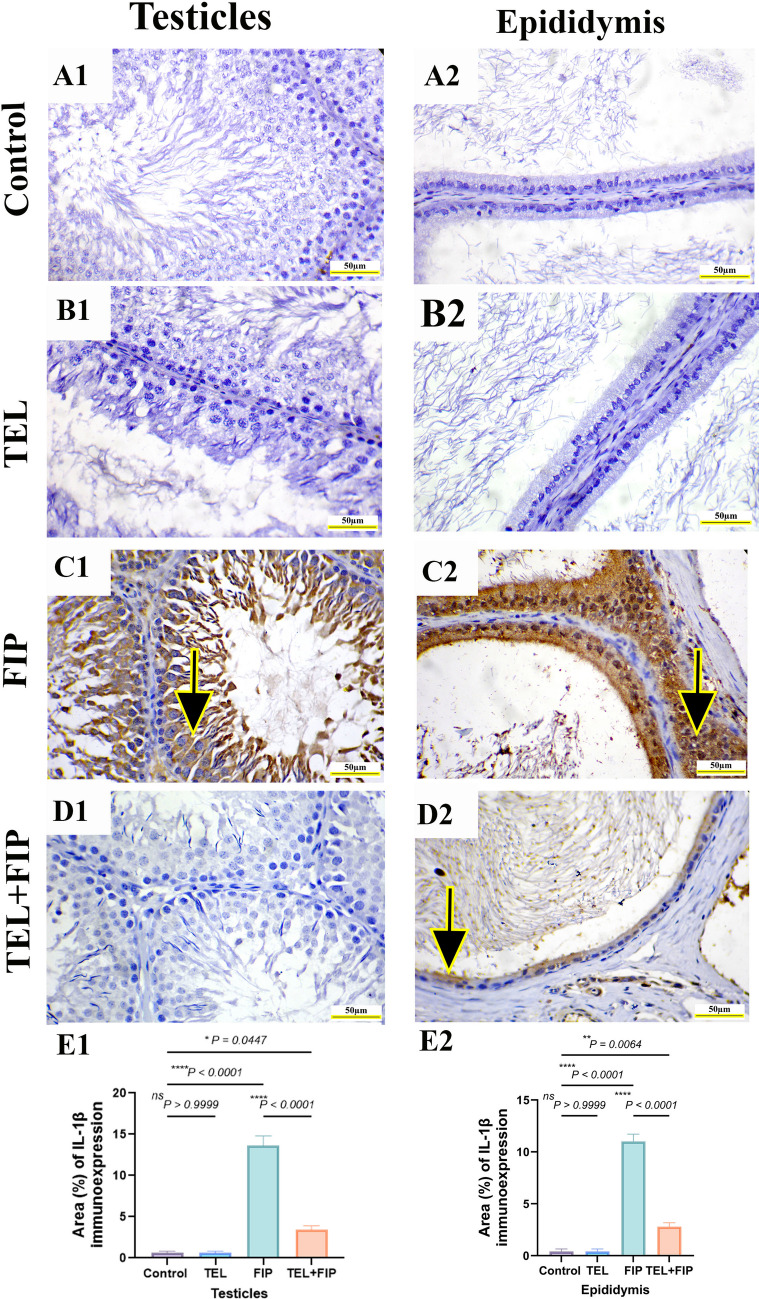
Effect of telmisartan (TEL) against fipronil (FIP) induced testicular and epididymal injury in rats. Immunohistochemical staining of interleukin-1*β* (IL-1*β*). Scale bar = 50 µm. (**A1**&**A2**) Control group showing negative IL-1*β* immunoreactivity in spermatogenic cells and epidydimal epithelium, (**B1**&**B2**) TEL treated group, (**C1**&**C2**) FIP group showing marked IL-1*β* immunoreactivity in spermatogenic cells and epidydimal epithelium, (**D1**&**D2**) TEL + FIP treated group showing mild to negative IL-1*β* immunoreactivity in spermatogenic cells and epidydimal epithelium and (**E1**&**E2**) Semi-quantitative analysis of IL-1*β* area % in the testicular and epididymal tissues from various experimental groups. Values presented as M ± SEM were assessed using a one-way ANOVA test, followed by Tukey’s post hoc test. *p < 0.05, **p < 0.01, ***p < 0.001, ****p < 0.0001

IL-1*β* immunohistochemical reactivity in rats’ testicles and epididymis revealed negative expression in spermatogenic cells and epididymal epithelium in control and TEL-treated animals (Fig. 10A1, B1, A2, and B2). Conversely, FIP-treated animals showed a strong IL-1*β* immunoreactivity in seminiferous tubules and epididymal epithelium (Fig. 10C1 and C2). TEL + FIP-treated animals revealed weak to negative IL-1*β* immunoreaction in the seminiferous tubules and epididymal epithelium (Fig. 10D1 and D2). Semi-quantification of IL-1*β* area% in the testicles and epididymis exhibited a significantly higher immunoreactivity of IL-1*β* in the FIP-treated animals relative to control and TEL-treated animals. This high immunoreactivity was substantially downregulated in the TEL + FIP-treated animals compared to FIP in both testicles and epididymis (Fig. 10E1 and E2).

### Effect of telmisartan on Nrf2 and HO-1 mRNA expressions

**Fig. 11:**
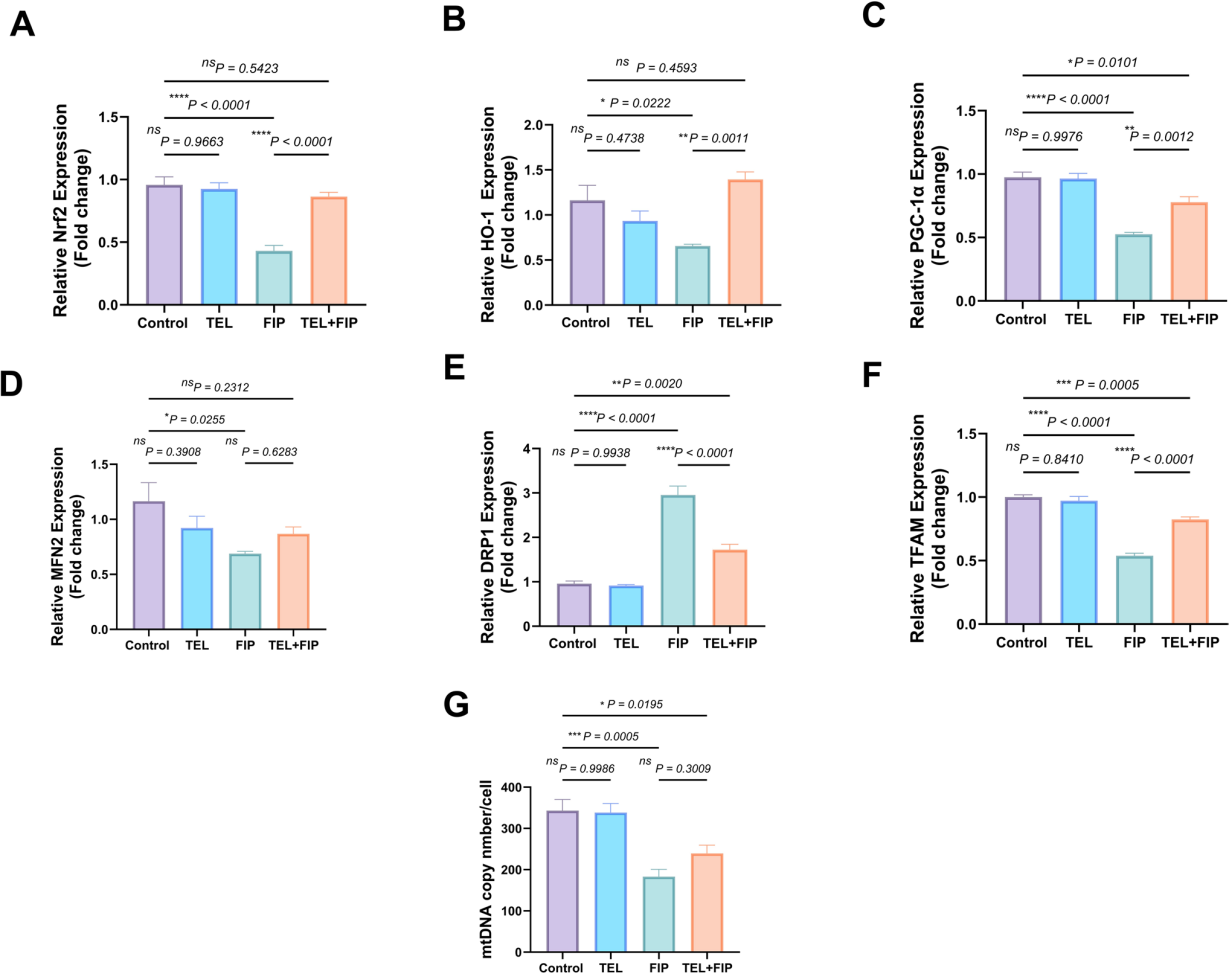
Effect of telmisartan (TEL) against fipronil (FIP) on mRNA expression induced testicular and epididymal injury in rats. (**A**) Nuclear factor erythroid 2-related factor 2 (Nrf2) mRNA transcript expression: (**B**) Heme oxygenase (HO)−1mRNA transcript expression. (**C**) Peroxisome proliferator-activated receptor-*γ* coactivator 1-*α* (PGC1*α*) mRNA transcript expression. (**D**) Mitofusin-2 (MFN2) mRNA transcript expression. (**E**) Dynamin-related protein 1 (Drp1) mRNA transcript expression. (**F**) Mitochondrial transcription factor A (TFAM) mRNA transcript expression. (**G**) Mitochondrial DNA (mtDNA) mRNA transcript expression. Values presented as M ± SEM were assessed using a one-way ANOVA test, followed by Tukey’s post hoc test. *p < 0.05, **p < 0.01, ***p < 0.001, ****p < 0.0001

No significant differences were observed in the gene expression of Nrf2 and HO-1 mRNA transcripts between TEL and control rats. However, the FIP-treated group significantly decreased the gene expression of Nrf2 and HO-1 mRNA transcripts by 55.11% and 43.5% in testicular tissue compared to the control group. The co-treatment of FIP-treated rats with TEL showed a significant upregulation in Nrf2 and HO-1 mRNA transcript gene expression by 100% and 112.5%, respectively, relative to FIP-treated animals (Fig. 11A and B).

#### Effect of telmisartan on mitochondrial dynamics-related genes

Regarding mitochondrial fission, the TEL group showed no considerable alterations in gene expression of PGC-1*α*, MFN2, and DRP1 mRNA transcript compared to the control group. Furthermore, FIP-intoxicated rats significantly declined (40.89% and 45.99%, respectively) MFN2 and PGC-1*α* mRNA transcripts, and upregulated DRP1 mRNA transcripts (208.6%) compared to the control rats. Additionally, TEL + FIP-treated rats did not reveal any considerable changes in testicular gene expression of MFN2 mRNA transcript, but significantly elevated PGC-1α mRNA transcript by 47.9% compared to the FIP-intoxicated group. The co-treatment of FIP with TEL significantly downregulated (41.75%) the testicular gene expression of DRP1 mRNA transcript compared to the FIP-intoxicated group (Fig. 11C-E).

Figures 11F and G show that TFAM and mtDNA expression show non-significant changes between control and TEL-treated groups. At the same time, TFAM and mtDNA expressions in the FIP-intoxicated group revealed a significant decrease (46.2 and 46.62%) compared to the control rats. On the other hand, the TEL + FIP co-treated group showed a significant increment in TFAM gene expression (53.16%) compared to the FIP-intoxicated group. Meanwhile, mtDNA gene expression in the TEL + FIP co-treated group revealed no change compared to the FIP-intoxicated group.

## Discussion

Chemical insecticides are widely used all around the world. Numerous factors, including pesticide residue on food, tainted tap water, industrial exposure, repellents, and use against fleas and ticks, can cause harm to both humans and animals 1 (Chagnon et al. 2015). FIP is considered a broad-spectrum insecticide widely utilized to manage veterinary, public health, and agricultural insects in Egypt (Noaishi et al. 2021). The usage of FIP is noticeably amplified as it serves as a substitute for old-fashioned insecticides such as organophosphates and pyrethroid pesticides (Abdel-Mobdy et al. 2023). The initiation of oxidative damage might be allied to the pathway of pesticide-caused testicular dysfunction (Tohamy et al. 2021). Thus, our study aims to explore the protective efficacy of TEL against FIP-mediated testicular damage by regulating oxidative damage (Nrf2, HO-1, and GSH) and mitochondrial dynamics (PGC-1*α*, MNF2, DRP1, TFAM, and mtDNA).

Additionally, testosterone levels in the serum are regarded as an effective diagnostic marker for optimum testicular function (Sharpe et al. 1992). Our study showed that FIP administration directly affected male rats’ fertility. That was confirmed by a noteworthy decrease in relative testicular weight, sperm count, and motility with sperm abnormalities. Likewise, rats that received FIP only exhibited a substantial decline in serum testosterone, FSH, and LH levels. This may be attributed to the inhibitory effect of FIP on the secretion of pituitary gonadotropins (FSH and LH), thus, the testosterone biosynthesis, which stops spermatogenesis (Saleh et al. 2023). Normal spermatogenesis and preserving the typical structure of seminiferous tubules require normal testosterone levels (Sharpe et al. 1992). The dropped LH level may also occur due to disrupting the hypothalamic-pituitary axis negative feedback control (Kovacs et al. 1997). Moreover, FIP could directly challenge testosterone and dihydrotestosterone for androgen receptors, thereby reducing testosterone synthesis (Kavlock and Cummings 2005). Histopathological examination corroborated these results, in which FIP resulted in interstitial edema, degeneration, and sloughing of germinal epithelium with the formation of multinucleated giant cells. Furthermore, FIP severely deteriorated Cosentino's and Johnson's scores. These results parallel previous reports (Saleh et al. 2020; Tohamy et al. 2021). Also, Tohamy et al. (2021)) stated that oral administration of FIP (20 mg/kg b.wt.) for 60 days caused a substantial decline in sperm count and progressive motility with an upsurge of the sperm abnormalities in male rats, deterioration in testicular histoarchitecture. Also, our findings are consistent with those of Saleh et al. (2023), who recorded a noteworthy decrease in serum testosterone and LH levels in rabbits that received FIP (17.7 mg/kg b.wt.) for four weeks. These results verified that FIP alters the anterior pituitary's ability to generate FSH, LH, and testosterone by initiating oxidative stress in the Leydig cells.

Stimulation of AT1R by angiotensin II, the key active element of the renin-angiotensin system that causes oxidative stress, inflammation, and apoptosis (Wolf 2000). The selective AT1R blocker, TEL, displayed significant antioxidant and anti-inflammatory characteristics and motivated the body's antioxidant defenses (Ceriello et al. 2007; Eslami et al. 2014). The present study exhibited that TEL ameliorated FIP-induced testicular dysfunction. Results showed that the concurrent treatment of TEL with FIP significantly improved the relative testicular weight, sperm count, and motility and decreased sperm abnormalities. This confirmed the defensive effects of TEL on the function of Leydig cells and steroidogenesis. The co-treatment of TEL with FIP also increased serum testosterone, FSH, and LH levels. This may be credited to TEL's substantial antioxidant and anti-inflammatory characteristics (Sato-Horiguchi et al. 2012). Better Cosentino and Johnson scores, TEL also improves testicular damage from FIP, indicating potential protection against FIP-related injury. These findings are consistent with prior studies on TEL's efficacy against testicular damage from toxins, arsenic (Fouad et al. 2015), cadmium (Fouad and Jresat 2013), and irradiations (Ran et al. 2023). But the effect of TEL against FIP-induced testicular injury mediated by mitochondrial damage has not been previously studied to the best of our knowledge.

Physiologically, the organism can protect itself against ROS via its reserves of antioxidant enzymes comprising GPx, glutathione reductase (GR), and CAT (Halliwell and Gutteridge 2015). Oxidative stress is the imbalance between ROS production and the activity of the antioxidant system that stimulates lipid peroxidation (Sies 1986). Our study reported that FIP administration to male rats significantly increased testicular oxidative stress. Results revealed an upturn in the testicular MDA and GSSG levels and a decline in GSH levels, CAT, and GPx activities. These results indicate that FIP-induced oxidative stress increases the prevalence of free radical production and lipid peroxidation. Those of Seydi et al. (2021) supported these outcomes and suggested the role of FIP to induce oxidative damage by producing more ROS. This might be ascribed to the capacity of FIP to trigger oxidative stress through diminishing the antioxidant system (the activity of CAT and GSH levels) in animals (Kartheek and David 2018; Mossa et al. 2015; Noaishi et al. 2021). These results follow Mazzo et al. (2018), who reported a substantial decrease in GSH levels and CAT and GPx activities in the testicles of male rats that received FIP (5 mg/kg) for 14 days. Also, come in consistent with Tohamy et al. (2021), who reported a substantial increase in the level of testicular MDA with a decline in GSH level after FIP-intoxication in male rats. They explained the decrement of CAT activity to the down-regulatory action of FIP on the mRNA expression of this enzyme. These findings support those of Abdel-Mobdy et al. (2023), who reported a significant rise in the hepatic MDA level with a drop in GSH level and CAT activity in FIP-intoxicated male albino rats (1/30 acute oral toxicity LD 50 of FIP). Moreover, Maher et al. (2024) stated a diminution in GSH content, CAT, and GPx activity with a significant upturn in MDA content in the brain homogenate of male albino rats given FIP (12.6 mg/kg b.wt.) over four weeks.

However, the co-treatment with TEL effectively reduced testicular MDA and GSSG content, increased CAT and GPx activities, GSH content, and GSH/GSSG ratio compared to the FIP-intoxicated group. This could be clarified by the direct antioxidant effect of TEL and its ability to activate the endogenous antioxidant defenses (Eslami et al. 2014). Moreover, Kuwashiro et al. (2011) and Sato-Horiguchi et al. (2012) specified that TEL mitigates lipid peroxidation and inhibits depletion of the antioxidant defense systems. Additionally, angiotensin II increases superoxide radical production, reducing nitric oxide levels and causing oxidative stress, increasing MDA content, inhibiting SOD action, and lowering tissues' GSH content (Goyal et al. 2011). Thus, TEL's capacity to reduce oxidative damage and improve indicators of oxidative stress in tissues might be attributed to its AT1R blocking activity and its interference with angiotensin II action (Pacher et al. 2005). These results harmonized with those of Fouad et al. (2015), who reported a substantial increase in testicular GSH levels with a significant decline in the level of MDA in rats treated with TEL against arsenite-intoxicated rats. Moreover, Gowda et al. (2022) mentioned that TEL (10 mg/kg b.wt. orally for 60 days) administration to Wistar rats improved arsenic-induced aortic dysfunction by increasing the aortic CAT, GPx activity, and GSH content with a reduction in the MDA content. Also, Abd-Eltawab et al. (2023) found that diabetic male albino rats treated with TEL (8 mg/kg/day for one month) showed a significant decrease in MDA content and a rise in GSH content in their skeletal muscles.

Regarding the pro-inflammatory cytokines, the current data revealed a noteworthy upsurge in the testicular pro-inflammatory cytokines’ levels (TNF-*α* and IL-1*β*) in the FIP-treated group. TNF-*α*is a major cytokine involved in inflammation and the immunological response (Lebda et al. 2018). These outcomes support those of Seydi et al. (2021), who postulated that FIP caused tissue inflammation by boosting the production of free radicals. Our findings align with Tohamy et al. (2021) and Maher et al. (2024), who reported a considerable rise in the testicular TNF-*α* of male rats intoxicated with FIP. The current data showed that the mixed treatment of FIP- FIP-intoxicated rats with TEL significantly reduced the testicular IL-1*β* and TNF-*α*levels. This could be illuminated by the prominent anti-inflammatory activity of TEL, which is explained by a decrease in inflammatory cytokines and chemokines levels as reported by Takagi et al. (2013) or through suppression of NF-*κ*B gene activation (Sugiyama et al. 2013). Also, Fouad et al. (2015) found that TEL (10 mg/kg/day) treatment considerably declines the expression of TNF-*α*in seminiferous tubule cells in rats intoxicated with arsenic. Similarly, Abdelhamid et al. (2021) and Gowda et al. (2022) declared that TEL co-treatment (10 mg/kg/day) decreased the content of hepatic and aortic IL-1*β* and TNF-*α*compared to alcoholic mice and arsenic-intoxicated rats, respectively. Maher et al. (2024) also observed a notable decrease in TNF-*α* levels in the skeletal muscles of diabetic male albino rats after TEL (8 mg/kg/day) treatment for four weeks.

The Nrf2 transcription factor stimulates the antioxidant response element via binding to the antioxidant cascade (Satta et al. 2017). Nrf2 promotes fatty acid peroxidation, mitochondrial respiration, and biosynthesis of PGC-1*α* and purine. It binds with co-activator PGC-1*α*to preserve the oxidative equilibrium and mitochondrial biogenesis in mitochondria and cytoplasm (Renu and Gopalakrishnan 2019). Nrf2 guards against excess free radicals and oxidative imbalance by upregulating transcription of cyto-defensive genes such as HO-1. HO-1 supports the antioxidant cascade by catalyzing heme's degradation into bilirubin, which scavenges peroxyl radicals and inhibits lipid peroxidation Abdel-Daim et al. 2019). The present study exhibited that FIP declined testicular Nrf2 and HO-1 expression in male albino rats compared to control rats. These results agree with Sakr et al. (2018) and Uzunhisarcikli et al. (2023), who explained that FIP downregulated the renal mRNA transcription of Nrf2 and HO-1 genes compared to the control group. Likewise, FIP uncouples oxidative phosphorylation, resulting in ATP depletion, ROS elevation, and downregulation of mRNA expression of Nrf2 (Abdel-Daim et al. 2019). Furthermore, in Wistar male albino rats, the neurotoxic effect of FIP was demonstrated by Maher et al. (2024) through Nrf2 downregulation. Similarly, they are consistent with the findings of Li et al. (2020), who reported that FIP decreased the expression of hepatic Nrf2 in Nile tilapia. Therefore, Oxidative redox induced by FIP could alter the antioxidant defense system and destroy cellular macromolecules, such as lipids, DNA, and proteins (Khan et al. 2015). In contrast, the co-administration of TEL stimulated the upregulation of Nrf2 and HO-1 mRNA expressions against FIP-intoxicated rats. Our results harmonized with those of Antar et al. (2022), who mentioned that in diabetic rats, the TEL treatment (5 and 10 mg/kg b.wt. for 8 weeks) upregulated the renal Nrf2 and HO-1. According to Saber et al. (2019) TEL also improves rats' colitis caused by dextran sodium sulfate by controlling the Nrf2/NF-*κ*B pathway. In addition, Abdelhamid et al. (2021) stated that TEL (10 mg/kg/day) considerably enhanced the expression of Nrf-2, PPAR-*γ*, and HO-1 against alcoholic liver disease. Moreover, TEL caused a marked elevation in HO-1 expression in cells against lipopolysaccharides (Choe et al. 2019). Similarly, TEL (5 mg/kg/day, oral) modulated NF-*κ*B and Nrf2 signaling pathways in mouse cuprizone-induced demyelination and behavioral impairment (Abd El Aziz et al. 2021). Additionally, TEL increased mRNA or protein expression of testicular Nrf2 and HO-1 in diabetic rats (Guo et al. 2016). Finally, these studies assured that TEL could reduce inflammation and oxidative stress in rats while upregulating Nrf2/HO-1 signaling.

PGC-1*α*is a transcriptional activator that controls gene transcription and contributes to mitochondrial energy biogenesis, homeostasis, and fatty acid and glucose oxidation (Cheng et al. 2018). In redox or inflammation, the PGC-1*α*expression and activity decreased, leading to the downregulation of antioxidant genes, resulting in oxidative imbalance (Abu Shelbayeh et al. 2023). In our study, FIP administration dramatically declined PGC-1*α*gene transcription, indicating that FIP promoted testicular mitochondrial dysfunction and oxidative stress. Similarly, Liu et al. (2017) found that FIP exposure decreased the expression level of PGC-1*α*and elevated the intracellular level of ROS, with a decrease in the ATP level in testicular Sertoli cells. This is consistent with Kong et al. (2010), who declared that PGC-1*α*acts as an ROS suppressor and enhances mitochondrial metabolic activity to alleviate lead-induced energy metabolic disorders. Additionally, Singh et al. (2016) stated that PGC-1*α* has a fundamental role in reducing ROS, mitochondrial biogenesis, energy metabolism, and mitochondrial fatty-acid oxidation. Finally, PGC-1*α*plays a crucial role in testicular metabolism impairment as it co-activates several nuclear transcriptors that regulate the expression of mitochondrial proteins (Liu et al. 2017). Meanwhile, our study declared a marked elevation in the expression of PGC-1*α*mRNA transcription in the FIP-intoxicated group co-treated with TEL. These results agree with Shiota et al. (2012), who revealed that TEL raised the mRNA levels of PGC1 in the skeletal muscle of obese mice. Also, Ray et al. (2024) demonstrated that TEL activated mitochondrial biogenesis, elevated PGC1*α*, MFN1, and ATP levels, with improved mitochondrial processes against Parkinson's disease in mice. In addition, our findings follow Sanchis-Gomar and Lippi (2012), who discussed that TEL elevated the levels of mitochondrial PGC-1*α*expression in adipose tissue. Additionally, TEL protects against obesity by boosting muscle fatty acid oxidation and activation of PGC1 (Takeuchi et al. 2013).

GTPases in the outer mitochondrial membrane, MFN1 and MFN2, mediate mitochondrial fission in sperm development in mammals. Male sterility in mice is caused by abnormalities in germ cell development caused by the loss of MFN1 or MFN2 alone (Miao et al. 2021). DRP1 is a GTPase that catalyzes this fission. DRP1 plays a role in cell death that is dependent on the mitochondria. FIP targets Mitochondria, where the dysfunction of mitochondria contributes to FIP-induced toxicity. Commonly, MFN2 deficiency negatively affects mitochondrial functions during spermatogenesis and meiosis. In the current study, the exposure to FIP decreased MFN2 mRNA transcription while increasing DRP1, indicating that FIP impaired energy metabolism and led to mitochondrial dysfunction and apoptosis. Our findings came following Sakr et al. (2018), who stated that FIP disrupts the permeability of mitochondrial membranes and downregulates pro-survival genes, causing cell apoptosis. Also, Ki et al. (2012) declared that exposure to FIP altered mitochondrial membrane potential and elevated free radical generation, LPO, inducing apoptosis of spermatozoa in a dose-dependent manner. Moreover, Park et al. (2016) concluded that cell death and cytotoxicity of FIP is primarily due to the excess generation of ROS and the decrease of DNA integrity of spermatozoa. The co-treatment of FIP-intoxicated rats with TEL alleviated the testicular alterations in MFN2 and DRP1 mRNA gene transcription to nearly the control level and markedly increased MFN2 mRNA gene transcription, while reducing DRP1 mRNA gene transcription as compared to FIP- FIP-intoxicated rats. The prominent anti-apoptotic activity of TEL may explain this. TEL was reported to have various signaling pathways in cancer cells, exhibiting anti-proliferative, anti-apoptotic effects (Hadjiyianni and Kalali 2023). Also, it has been shown that TEL has beneficial antioxidant activity, inhibits apoptotic response, and suppresses DRP1 gene activity (García-Sánchez et al. 2020).

Testicular mitochondria are the fundamental source of ATP synthesis and sperm quality to safeguard testicular activities (Huang et al. 2020). Meanwhile, mitochondria are the principal targets of redox-induced destruction of mtDNA and mitochondrial respiratory inefficiency (Zorov et al. 2014). Mitochondrial biogenesis and autophagy regulate the mtDNA copy number gene coding (Gaziev et al. 2014). According to our investigation, the administration of FIP to male albino rats markedly declined mtDNA compared to the control group—findings of Souders et al. (2021) and Vidau et al. (2011) suggested that FIP decreased neuronal mtDNA because it is a Mito toxicant, a potent uncoupler of oxidative phosphorylation, and increases oxygen consumption. Furthermore, FIP caused sperm toxicity in rats via DNA fragmentation, decreasing mtDNA, chromatin destruction in sperm, mitochondrial dysfunction, and apoptosis of the spermatozoa 7 (Khan et al. 2015). Moreover, Sayed et al. (2019) demonstrated that FIP induced the production of peroxisomes and damaged both DNA and mitochondria in rats’ hepatic tissues. TFAM is a key activator of mammalian mitochondrial transcription and enhances mtDNA maintenance, besides acting as a regulator of mtDNA copy number. TFAM alleviates oxidative damage to mtDNA (Picca et al. 2014). Compared to control rats, the rats that received FIP exhibited a substantial decrease in mtDNA and TFAM mRNA transcripts. Similarly, Rantanen et al. (2001) observed significant reductions in mitochondrial TFAM protein levels associated with the appearance of testis-specific TFAM mRNA isoforms. Finally, TFAM is nucleus-encoded, and its expression is regulated by nuclear respiratory factors NRF-1 and NRF-2 (Piantadosi and Suliman 2006). On the other hand, the present study also showed that TEL countered FIP, inducing a significant decline in TFAM and mtDNA gene expression. In agreement with our results, Sugimoto et al. (2006) recorded that TEL increases TFAM transcription in rats fed high-fructose and high-fat diets. Furthermore, another study showed that TEL improved mitochondrial dysfunction in endothelial damage (Nozaki et al. 2009). At the same time, Takeuchi et al. (2013) observed that mtDNA copy number did not alter after administration of TEL for 2 days, showing that mitochondrial biogenesis might not affect mitochondrial function in human and mouse wild-type vascular smooth muscle cells.

## Conclusion

A novel understanding of TEL administration at 10 mg/kg/day may protect against FIP-induced testicular injury through antioxidant, anti-inflammatory, and mitochondrial-enhancing effects. This is mediated by upregulating Nrf2/HO-1/PGC-1α expression and PCNA immunoreactivity while downregulating IL-1*β*, TNF*α*, and DRP1 mRNA transcripts. TEL preserved testicular and epididymal structure, increased testicular weight, enhanced serum testosterone levels, restored sperm count and motility, and reduced abnormalities. This study is the first to provide mechanistic insights into TEL’s potential preventative effects on testicular damage from FIP, presenting a strategy for addressing male infertility. Further research is needed to explore TEL’s efficacy in mitochondrial fusion and fission related to male infertility.

In conclusion, this groundbreaking study sheds light on the potential of TEL as a powerful ally in the fight against testicular injury induced by FIP. By unveiling its multifaceted protective effects ranging from antioxidant and anti-inflammatory properties to enhance the mitochondrial function. TEL presents a promising therapeutic avenue for addressing male infertility. The ability to preserve testicular architecture, boost testosterone levels, and restore vital sperm parameters highlights its significant role in male reproductive health. As we stand on the brink of new possibilities, further research will be essential to harness TEL’s capabilities entirely, especially in understanding its influence on mitochondrial dynamics. This could pave the way for innovative strategies to not only prevent testicular damage but also enhance fertility outcomes, offering hope to many in need.

## Data Availability

All source data for this work (or generated in this study) are available upon reasonable request.

## Abbreviations

FIP: Fipronil
WHO: World Health Organization
GABA: Gamma-aminobutyric acid
GSH: Glutathione
MDA: Malondialdehyde
RAS: Renin-angiotensin system
AT1R: Angiotensin 1 receptor
ACE: Angiotensin-converting enzyme
ARBs: Angiotensin receptor blockers
TEL: Telmisartan
PPAR-*γ*: Peroxisome proliferator-activated receptor-*γ*
GSK3*β*: Glycogen synthase kinase-3 beta
PGC1α: Peroxisome proliferator-activated receptor-γ coactivator 1-*α*
NF-KB: Nuclear factor-kappa B
MFN1: Mitofusin 1
MFN2: Mitofusin 2
DRP1: Dynamin-related protein 1
Fis1: Fission 1
PPARGC1β: Peroxisome proliferator-activated receptor-*γ* coactivator 11*β*
TFAM: Mitochondrial transcription factor A
mtDNA: Mitochondrial DNA
Nrf2: Nuclear factor erythroid 2–related factor 2
HMOX1: Heme oxygenase-1
IACUC: Institutional Animal Care and Use Committee
CAT: Catalase
GPx-1: Glutathione peroxidase-1
FSH: Follicular-stimulating hormone
LH: Luteinizing hormone
tGSH: Total glutathione
GSH: Reduced glutathione
GSSG: Oxidized glutathione
TNF-α: Tumor necrosis factor-alpha
IL-1*β*: Interleukin-1*β*
PBS: Phosphate-buffered saline
PCNA: Proliferating cell nuclear antigen
ANOVA: Analysis of variance
GR: Glutathione reductase
ROS: Reactive oxygen species
